# A Systemic Selective Modified mRNA Delivery Platform for Preventing Chemotherapy‐Induced Cardiotoxicity

**DOI:** 10.1002/advs.202510543

**Published:** 2026-01-16

**Authors:** Jimeen Yoo, Gayatri Mainkar, Matteo Ghiringhelli, Bernardo Gindri Dos Santos, Magdalena M. Żak, Keerat Kaur, Ann Anu Kurian, Matthew Adjmi, Segev Sharon, Eftychia Markopoulou, Lucia Žigová, Daniela Hirsch, Sydney Pessin, Rachel Hanan, Juan Luis Escano Abreu, Niki Brisnovali, Nicole Dubois, Leigh Goedeke, Mengcheng Shen, Lior Zangi

**Affiliations:** ^1^ Cardiovascular Research Institute Icahn School of Medicine at Mount Sinai New York New York USA; ^2^ Department of Genetics and Genomic Sciences Icahn School of Medicine at Mount Sinai New York New York USA; ^3^ Black Family Stem Cell Institute Icahn School of Medicine at Mount Sinai New York New York USA; ^4^ Cell, Development/Regenerative Biology Icahn School of Medicine at Mount Sinai New York New York USA; ^5^ Department of Medicine, Division of Cardiology Center for Cardiovascular Research Washington University Saint Louis Missouri USA

**Keywords:** cardiac‐selective delivery, modified mRNA, Dox cardiotoxicity

## Abstract

Doxorubicin (Dox) is a widely employed chemotherapeutic agent, but its use is clinically limited by dose‐accumulative cardiotoxicity. More specifically, Dox induces oxidative stress and causes pro‐apoptotic ceramide accumulation in cardiomyocytes (CMs). Acid ceramidase (AC) modified mRNA (modRNA) has been shown to reduce ceramide levels and protect the heart following ischemic injury; however, therapeutic modRNA applications have been hindered by the need for invasive delivery. Here, we present a platform for minimally intrusive transmission of modRNA to the heart. This CM‐selective modRNA translational system (cmSMRTs) is encapsulated in lipid nanoparticles for intravenous (IV) delivery to enable systemic administration with high cardiac selectivity via microRNA‐guided translational control (miR143 and miR122) to suppress off‐target expression in other tissues, including tumors. In vitro, AC treatment preserved sarcomere structure, calcium handling, and mitochondrial function in Dox‐treated human induced pluripotent stem cell (iPSC)‐derived CMs. Moreover, weekly IV delivery of this modRNA prevented cardiac dysfunction, fibrosis, and atrophy in chronic Dox‐induced cardiotoxicity models. Notably, this cardioprotection is achieved without either compromising Dox's anti‐tumor efficacy or producing overall toxicity. These findings establish cmSMRTs 143‐122 as a minimally invasive, cardiac‐selective mRNA therapy platform with strong potential to prevent chemotherapy‐induced cardiotoxicity.

## Graphical abstract

In Yoo and Mainkar et al., we present a minimally invasive, CM‐selective modRNA delivery system encapsulated in lipid nanoparticles for intravenous (IV) administration. This platform enables selective cardiac translation of therapeutic modRNA but suppresses expression in off‐target tissues, including tumors. We show that our platform can prevent Dox‐induced cardiotoxicity without either compromising Dox's anti‐tumor efficacy or producing overall toxicity.

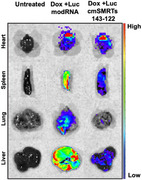

## 1 Introduction

As cancer diagnosis and treatment advance, the number of cancer survivors continues to grow, as does the number of patients experiencing chemotherapy‐related side effects [1]. Anthracyclines such as doxorubicin (Dox) form a class of chemotherapeutic agents that have become a treatment mainstay for solid and hematologic malignancies, including prostate or breast cancer and childhood leukemias. However, the use of anthracyclines limited by cardiotoxicity, which can cause both acute and delayed systolic and diastolic dysfunction and, ultimately, heart failure [2]. The incidence of Dox‐induced cardiomyopathy ranges from 3% to 57% depending on key clinical variables, including age, cumulative dose, combined therapies, and preexisting cardiovascular condition [3].

At the cellular level, the cardiotoxic effects of Dox have been attributed to multiple mechanisms, including oxidative stress, DNA damage, ferroptosis, aberrant calcium handling, mitochondria dysfunction, and fibrosis [4, 5, 6]. Additionally, ceramide accumulation is an often‐overlooked cytotoxic consequence of Dox treatment [7, 8, 9]. Ceramide, a bioactive sphingolipid, serves as an effector molecule in apoptotic signaling pathways [10]. Because ceramide accumulation enhances cell death, it is a desirable outcome in cancer treatment but deleterious in cardiomyocytes (CMs) [11, 12]. Current clinical strategies seek to mitigate Dox cardiotoxicity by employing liposomal formulations to enhance tumor‐selective delivery and by co‐administering dexrazoxane to chelate iron and suppress ROS generation. Yet, 9% of cancer survivors still develop Dox cardiotoxicity; therefore, more effective therapies are needed to prevent or mitigate this off‐target consequence [13, 14, 15].

In humans, acid ceramidase (AC) protects against various diseases by catalyzing ceramide hydrolysis into sphingosine, which is subsequently phosphorylated to form sphingosine‐1‐phosphate (S1P), a bioactive lipid with anti‐apoptotic and cardioprotective properties [16, 17, 18]. Because it maintains a balance between ceramide and S1P, AC has emerged as a promising therapeutic candidate in diverse pathological contexts [19]. Notably, transient overexpression of AC has been shown to confer cardioprotection by modulating sphingolipid metabolism in a murine model of myocardial infarction (MI) [20].

Nucleoside‐modified messenger RNA (modRNA) therapies are a novel class of drugs that gained widespread attention following the success of COVID‐19 mRNA vaccines produced by Moderna and Pfizer‐BioNTech [21]. Beyond infectious disease, RNA therapeutics have shown great potential for treating cardiovascular disorders [22]. However, broader modRNA applications for cardiac diseases have been hampered by the lack of platforms that enable tissue‐ or cell type‐selective expression. In prior work, we demonstrated that incorporating microRNA (miR) recognition elements (for miR1 and miR208) into RNA constructs facilitates CM‐selective expression by leveraging endogenous differences in miR profiles across cardiac cell types [23, 24, 25, 26]. Here, we added two additional miR recognition elements (for miR143 and miR122) to reduce unwanted translation in organs other than the heart. This approach, together with encapsulation in self‐assembling lipid nanoparticles (LNPs), allowed us to create a superior, IV‐administered modRNA delivery tool, called cmSMRTs 143‐122, that selectively expresses genes in the heart. We then evaluated the therapeutic capacity of AC cmSMRTs 143‐122 in preventing Dox‐associated cardiotoxicity in C57VL/6 mice or mice bearing prostate or breast cancer tumors.

In summary, we here present a transformative therapeutic approach to impede chemotherapy‐induced cardiotoxicity through a tissue‐selective, minimally invasive modRNA delivery system that enables targeted organ protection without compromising the anti‐cancer efficacy of Dox treatment and without requiring open chest surgery and intramyocardial delivery.

## 2 Methods

### 2.1 Animals

All animal procedures were conducted in accordance with protocols approved by the Institutional Animal Care and Use Committee (IACUC) at the Icahn School of Medicine at Mount Sinai (protocol number IACUC‐2015‐0043). Male C57BL/6J and female Balb/c mice (8 weeks old) were obtained from Charles River Laboratories and housed under controlled conditions (22°C, 55% humidity) on a 12‐hour light/dark cycle with ad libitum access to food and water. After a one‐week acclimation period, mice were randomly assigned to experimental groups (*n *= 7,8 for modRNA expression biodistribution studies; *n* = 5,6 per group for Dox‐induced cardiotoxicity experiments). Neonatal CMs were isolated from 2‐day‐old C57BL/6 pups by using the Pierce Cardiomyocyte Isolation Kit, according to the manufacturer's instructions. Isolated CMs were seeded at a density of 5 × 10⁵ cells per well in 24‐well plates.

### 2.2 Dox Treatment

Doxorubicin hydrochloride (Dox; Sigma‐Aldrich, D1515) was dissolved in phosphate‐buffered saline (PBS, pH 7.4) to produce a 1 mg/mL stock solution. To protect it from light, the solution was wrapped in aluminum foil and stored at 4°C until use. Dox treatment was administered following previously established protocols, with cumulative doses ranging from 15 to 32 mg/kg over 4–6 weeks [27, 28]. This study employed a final cumulative dose of 15–20 mg/kg which was selected based on clinical relevance and widespread use in rodent models of chronic cardiotoxicity [29]. Male C57BL/6J or female Balb/c mice assigned to Dox groups received weekly intraperitoneal (IP) injections of Dox at 5 mg/kg for 3–4 weeks. Control animals received equal volumes of saline as a vehicle. Injection volumes were adjusted according to body weight and ranged from 100 to 120 µL. For in vitro experiments, hiPSC‐CMs and neonatal CMs were treated with 0.5 µm of Dox or equivalent volumes of PBS, as per previously established protocols [30].

### 2.3 Constructing IVT Templates and Synthesizing modRNA

ModRNAs were transcribed in vitro from plasmid templates (see Table S1 for plasmid ORF sequences), using a customized ribonucleotide mix containing GTP, ATP, CTP (Invitrogen), N1‐methylpseudouridine‐5′‐triphosphate (Trilink Biotechnologies), and CleanCap AG (3′ OM, Trilink Biotechnologies). In vitro transcription was performed as previously described [29]. The resulting modRNAs were purified using the Qiagen RNeasy Midi Kit, following the manufacturer's instructions. Purified RNA was then concentrated using Amicon Ultra 10K centrifugal filters (Millipore) and quantified using a Nanodrop spectrophotometer (Thermo Scientific). RNA size and integrity were assessed using an Agilent Bioanalyzer.

### 2.4 Differentiation of Human Induced Pluripotent Stem Cells (hiPSCs)

Human induced pluripotent stem cells (hiPSCs, derived from healthy adult fibroblasts) were differentiated into CMs by using an embryoid body (EB)‐based protocol. hiPSCs were maintained in mTeSR Plus medium and passaged every 3–4 days onto Geltrex‐coated plates. To initiate EB formation, cells were treated with 0.05 mm EDTA for 5 min or until detachment, collected by centrifugation (300 × g, 3 min), and resuspended in differentiation medium composed of RPMI (Gibco), 2 mm *L‐*glutamine (Invitrogen), 4 × 10^−4^ m monothioglycerol (MTG, Sigma), 50 µg/mL ascorbic acid (Sigma), and 150 µg/mL transferrin (Roche). On day 0, differentiation medium was supplemented with 2 ng/mL BMP4 and 3 µm Thiazovivin (Millipore Sigma). EBs were cultured in six‐well ultra‐low attachment plates (Corning) at 37°C in a hypoxic environment (5% CO_2_, 5% O_2_, 90% N_2_). On day 1, medium was refreshed with differentiation medium containing 20 ng/mL BMP4, 5 µg/mL bFGF, 20 ng/mL Activin A (R&D Systems), and 3 µm Thiazovivin. On day 3, medium was replaced with differentiation medium supplemented with 5 ng/mL VEGF and 5 µm XAV939 (Stemgent). On days 5 and 7, medium was refreshed with VEGF‐containing differentiation medium. From day 10 onward, cells were maintained in unsupplemented differentiation medium changed every four days.

### 2.5 Sarcomere Length and Organization Analysis

To assess sarcomeric integrity, differentiated hiPSC‐CMs were seeded onto 24‐well plates with coverslips pre‐coated with 1:200 diluted Matrigel (Corning). Following Dox treatment, cells were fixed and immunostained for cardiac troponin T (TNNT2) and sarcomeric α‐actinin (ACTN2). Z‐line morphology was analyzed using the Z‐line Detection algorithm to quantify z‐line length and sarcomere alignment angle across treatment groups [30]. Image analysis was performed using custom Python code (PyCharm 2024.1.4).

### 2.6 Calcium Imaging

Calcium transients were measured in day 25–30 hiPSC‐CMs cultured in MatTek dishes (35 mm, No. 1.0 coverslip, 20 mm glass diameter) pre‐coated with 1:200 diluted Matrigel (Corning). After a 3‐day recovery period, cells were incubated with 2 µm Fura‐2 AM (Thermo Fisher) for 15 min at 37°C in Tyrode's solution (Boston Bioproducts). This completely de‐esterified intracellular Fura‐2 and stabilized calcium dynamics before recording. Ca^2^⁺ imaging was performed under electrical stimulation (2 Hz, MyoPacer field stimulator, IonOptix), using the CytoCypher high‐throughput platform. Cells were alternately excited at 340 and 380 nm, and fluorescence emission was collected at 510 nm. The 340/380 nm ratio was used to quantify calcium transients over time. Recordings were captured with IonWizard 7.3 software (IonOptix) and analyzed using the Cytosolver Cloud Transient Analysis Software (Beta version, Cytocypher).

### 2.7 ModRNA Transfection

#### 2.7.1 In Vitro Transfection

AC or nGFP modRNAs were transfected into neonatal mouse CMs or hiPSC‐CMs by using Lipofectamine RNAiMAX (Life Technologies). Transfection complexes were prepared according to the manufacturer's instructions and added to cells cultured in DMEM supplemented with growth factors and 10% fetal bovine serum (FBS; Lonza).

#### 2.7.2 In Vivo Transfection

For IV injections, 75 µg of Luc, Cre, or AC modRNAs, with 25 µg nGFP or Cas 6 modRNAs at a 4:1 ratio (100 µg total), were encapsulated in LNPs and administered systemically. LNPs were formulated in‐house using the Ignite microfluidic mixing system (Precision NanoSystems). Each formulation contained the following lipids in ethanol: Lipid 5 (MedChemExpress, #HY‐138170), DSPC (Avanti Polar Lipids, #850365P), cholesterol (Avanti, #700000P), and DMG‐PEG2000 (Avanti, #880151P) at a ratio of 50:10:38.5:1.5. The aqueous phase consisted of modRNA in RNase/DNase‐free water and the organic phase consisted of the lipids dissolved in ethanol. Aqueous and organic phases were mixed at a 3:1 ratio by using the microfluidic platform. The resulting modRNA‐LNPs were diluted in PBS and dialyzed overnight at 4°C using dialysis cassettes (Thermo Fisher, #A52972). Final formulations were concentrated with Amicon Ultra 10K centrifugal filters (Millipore, #UFC9010) and adjusted to a final volume of 100–200 µL per injection. LNP quality was assessed by measuring particle size and polydispersity index (PDI) using a dynamic light scattering (DLS) particle size analyzer (Litesizer 500, Anton Paar). RNA encapsulation efficiency was determined using the Quant‐iT RiboGreen RNA Assay Kit (Invitrogen, #R11490), following the manufacturer's protocol.

#### 2.7.3 Bioluminescence Imaging (IVIS)

In vivo Luc expression was assessed at multiple time points by using the IVIS Spectrum Imaging System (NCRR S10‐RR026561‐01) at the Preclinical Small Imaging Core, Mount Sinai Medical Center. Mice were anesthetized with isoflurane (Abbott Laboratories), and *D*‐luciferin (150 mg/kg; Sigma) was administered intraperitoneally. Imaging was performed every 2 min until luminescence plateaued. Bioluminescence signals were quantified using Living Image software.

#### 2.7.4 Echocardiography

Cardiac function was assessed by transthoracic echocardiography using the Vevo 2100 system (VisualSonics) with a 40 MHz probe. Left ventricular systolic function was evaluated by measuring percent ejection fraction (EF) and fractional shortening (FS) from M‐mode images at the mid‐papillary level. %EF might vary due to strain‐, and sex‐based variability (C57BL/6 males or athymic nude mice male or Balb/c females), and because different tumor burden in the cancer models might impact cardiac function.

#### 2.7.5 RNA Isolation and qPCR

Total RNA was extracted using the Quick‐RNA Miniprep Kit (Zymo Research) and reverse‐transcribed using the iScript cDNA Synthesis Kit (Bio‐Rad) per the manufacturers’ instructions. Quantitative PCR was performed using the PerfeCTa SYBR Green FastMix (QuantaBio) on a Mastercycler Realplex 4 (Eppendorf). Gene expression was normalized to GAPDH, and relative fold changes were calculated using the ΔΔCt method. All primer sequences used are available in Table S2.

#### 2.7.6 RNA Sequencing and Analysis

Poly(A)‐selected RNA was prepared by the Epigenomics Core at Weill Cornell Medical College, using the Illumina mRNA‐Seq Sample Prep Kit. Libraries were sequenced using 50‐bp single‐end reads on the Illumina HiSeq 2000 platform. An average of 30 million reads per sample was obtained, with a mean quality score of 35.2. Reads were aligned to the mouse genome (mm10) by using STAR v2.5.3a. Gene counts were generated using the Partek E/M algorithm with the UCSC RefSeq 2017‐08‐02 annotation. Data normalization (TMM or RPKM) and differential gene expression analysis were performed using Partek Flow GSA. Gene ontology analysis was conducted using the Enrichr tool.

#### 2.7.7 Neonatal Mouse Cardiomyocyte Isolation

CMs from 2‐day‐old C57BL/6 pups were isolated by using the Pierce Cardiomyocyte Isolation Kit (Thermo Scientific, Catalog Number 88281). CMs were seeded at a density of 5 × 10⁵ cells per well in a 24‐well plate, following the kit's recommended protocol.

#### 2.7.8 Immunohistochemistry (IHC)

For tissue IHC, mouse hearts were perfused and fixed in 4% PFA overnight, followed by incubation in 30% sucrose at 4°C. Hearts were embedded in OCT, cryosectioned at 10 µm, and blocked in 5% donkey serum. Sections were incubated overnight at 4°C with primary antibodies against troponin I and GFP, followed by fluorescent secondary antibodies (Jackson ImmunoResearch) and Hoechst 33342 nuclear counterstain. For cell IHC, neonatal mouse CMs or hiPSCs were fixed in 4% PFA for 10 min, permeabilized with 0.1% Triton X‐100, and blocked with 5% donkey serum. Cells were incubated with primary antibodies, followed by fluorescent secondary antibodies and Hoechst 33342. To immunostain neonatal CMs for mCherry presence following modRNA treatment, cells were fixed on coverslips with 4% PFA for 15 min at room temperature, then washed three times with PBS with 0.1% Triton X‐100 (PBST). Cells were permeabilized in PBST for 7 min followed by overnight staining with primary antibodies. We used the recommended concentrations of sacromeric a‐actinin (Abcam, Ab9465), tdTomato (Sicgen, AB8181), and Vimentin (MA5‐11883, Invitrogen). The next day, slides were washed with PBST (five times, 4 min each) followed by incubation with a secondary antibody (Invitrogen, 1:200) diluted in PBST for 2 h at room temperature. To remove the secondary antibody, the samples were further washed with PBST (three times, 5 min each) and stained with Hoechst 33 342 (1 mg/mL) diluted in PBST for 7 min. After five 4‐minute washes with PBST, slides were mounted with mounting medium (Vectashield) for imaging. Stained slides were stored at 4°C. Images were acquired using a Zeiss fluorescence microscope at 20× magnification. All antibodies used are listed in Table S3.

#### 2.7.9 Mouse Ceramide (CER) Elisa Kit

After different treatments, mouse hearts were collected, homogenized, and added to a 96‐well plate pre‐coated with a Ceramide antibody (AFG Scientific). Standards and samples were incubated at 37°C for 40 min. Next, the plate was washed 5 times by the wash solution, HRP was added, and the plate was covered and incubated at 37°C for 40 min. After incubation, the plate was washed again, and Chromogen B was added. The plate was covered and incubated at 37°C for 20 min. Finally, a Stop solution was added to halt the reaction, and the absorbance was read at 450 nm to quantify the ceramide concentration.

#### 2.7.10 Masson's Trichrome Staining

Frozen OCT‐embedded heart sections were air‐dried and stained using the Masson's Trichrome protocol to assess cardiac fibrosis. Sections were pre‐treated with Bouin's solution, then stained sequentially with Weigert's Iron Hematoxylin, Biebrich Scarlet‐Acid Fuchsin, Phosphotungstic/Phosphomolybdic Acid, and Aniline Blue. Sections were differentiated in acetic acid, dehydrated in graded ethanol, cleared with xylene, and mounted with Permount. Fibrotic tissue was visualized by brightfield microscopy.

#### 2.7.11 Complete Blood Count (CBC) and Liver Enzyme Assays

Whole blood and serum were analyzed by the Mount Sinai Hospital Pathology Core. CBC was performed to assess lymphocytes, monocytes, neutrophils, eosinophils, and platelets. Serum liver enzyme levels—including alanine aminotransferase (ALT), aspartate aminotransferase (AST), alkaline phosphatase (ALP), and albumin (ALB)—were measured using the AU680 Chemistry System (Beckman Coulter). Assays were quality‐controlled using Westgard rules.

#### 2.7.12 Hematoxylin and Eosin (H&E) Staining

Paraffin‐embedded sections of heart, liver, spleen, and lung were air‐dried, rehydrated in PBS, and stained with hematoxylin for 2 min followed by eosin for 1 min. Slides were washed, dehydrated in ethanol and xylene, and mounted with Permount. Tissue morphology was evaluated by brightfield microscopy.

#### 2.7.13 Statistical Analysis

Statistical analyses were performed using GraphPad Prism. Comparisons between two groups were made using unpaired two‐tailed *t*‐tests. For multiple group comparisons, one‐way or two‐way ANOVA followed by Tukey's multiple comparison test was used, as specified in the figure legends. A *p*‐value < 0.05 was considered statistically significant. Data were presented as mean ± standard error of the mean (SEM). Statistical significance was indicated as follows: ****p < 0.0001, ***p < 0.001, **p < 0.01, *p < 0.05, ns = not significant.

## 3 Results

### 3.1 Engineering cmSMRTs 143‐122 for Systemic and Selective Delivery to the Heart

We previously developed a CM‐selective system that enables modRNA translation specifically in CMs (cmSMRTs). This system is composed of two modular constructs: an RNA‐binding protein modRNA (e.g., L7AE or Cas6) containing miR‐1 and miR‐208 recognition elements in its 3′UTR, and a gene‐of‐interest modRNA (e.g., luciferase [Luc]) bearing an L7AE or Cas6‐cleavable hairpin in its 5′UTR [26]. In CMs, which highly and exclusively express miR‐1 and miR‐208, the RNA‐binding protein is degraded, thereby allowing the gene of interest to be translated. In contrast, in non‐CMs, the RNA‐binding protein persists and inhibits gene translation by cleaving the hairpin structure (Figure S1a). We demonstrated that direct myocardial injection of this construct enables CM‐selective gene expression in mouse and pig models post‐MI [23, 25].

To achieve systemic delivery, we encapsulated Luciferase (Luc) cmSMRTs into self‐assembling LNPs containing Lipid 5 as the cationic lipid (Figure S1b). Lipid 5 is known to promote efficient encapsulation, improved endosomal escape, and favorable safety due to rapid systemic clearance [31]. IV injection of Luc‐modRNA or Luc cmSMRTs into adult C57BL/6 mice followed by IVIS imaging revealed that conventional modRNA generated broad organ‐wide expression, but cmSMRTs produced significantly less translation in the lung and spleen while maintaining expression in the heart and liver (Figure S1c–f). This residual liver signal is consistent with the known hepatic tropism of LNPs containing Lipid 5 [31]. We have focused our off‐target quantification on the liver, spleen, and lung because these are highly vascularized organs that accumulate LNPs due to high LNP uptake.

In order to further limit off‐target expression, we incorporated additional tissue‐specific miRNA recognition elements into the gene‐of‐interest modRNA. miR‐143 is highly expressed in immune and vascular smooth muscle cells, but its expression in CMs is restricted to the cardiac developmental stage [32, 33, 34, 35], whereas miR‐122 is liver‐specific and absent from the heart [36]. By integrating miR‐143 and miR‐122 recognition sequences into the 3’UTR of Luc modRNA, we created cmSMRTs 143, cmSMRTs 122, and cmSMRTs 143‐122, designed to suppress translation in the liver, lung, and spleen, while preserving cardiac gene expression (Figure S2a–f). We show that under normal conditions or after chemotherapy with Dox, cmSMRTs carrying miR143 had significantly decreased cmSMRTs expression in the lung (Figure S2d) and cmSMRTs carrying miR122 had reduced expression in the liver (Figure S2f). In addition, Dox treatment increases overall expression, compared to that seen during normal conditions, probably due to induced vascular permeability, which is a known side effect of Dox treatment [37] and may enhance LNP uptake in peripheral tissues. Therefore, we designed cmSMRTs carrying both miR143 and miR122 recognition elements (i.e., cmSMRTs 143‐122) to examine whether this approach would lessen unwanted translation in the lung and liver without compromising cmSMRTs expression in the heart (Figure S2c). While further testing our cmSMRTs’ ability to selectively express genes of interest in CMs and reduce translation in non‐CMs, we delivered nuclear mCherry as either cmSMRTs or modRNA into isolated neonatal cardiac cells (Figure S2g) and found that cmSMRTs transfection resulted in significantly less nuclear mCherry translation in non‐CMs (identified by vimentin which is a marker for non‐CMs).

**FIGURE 1:**
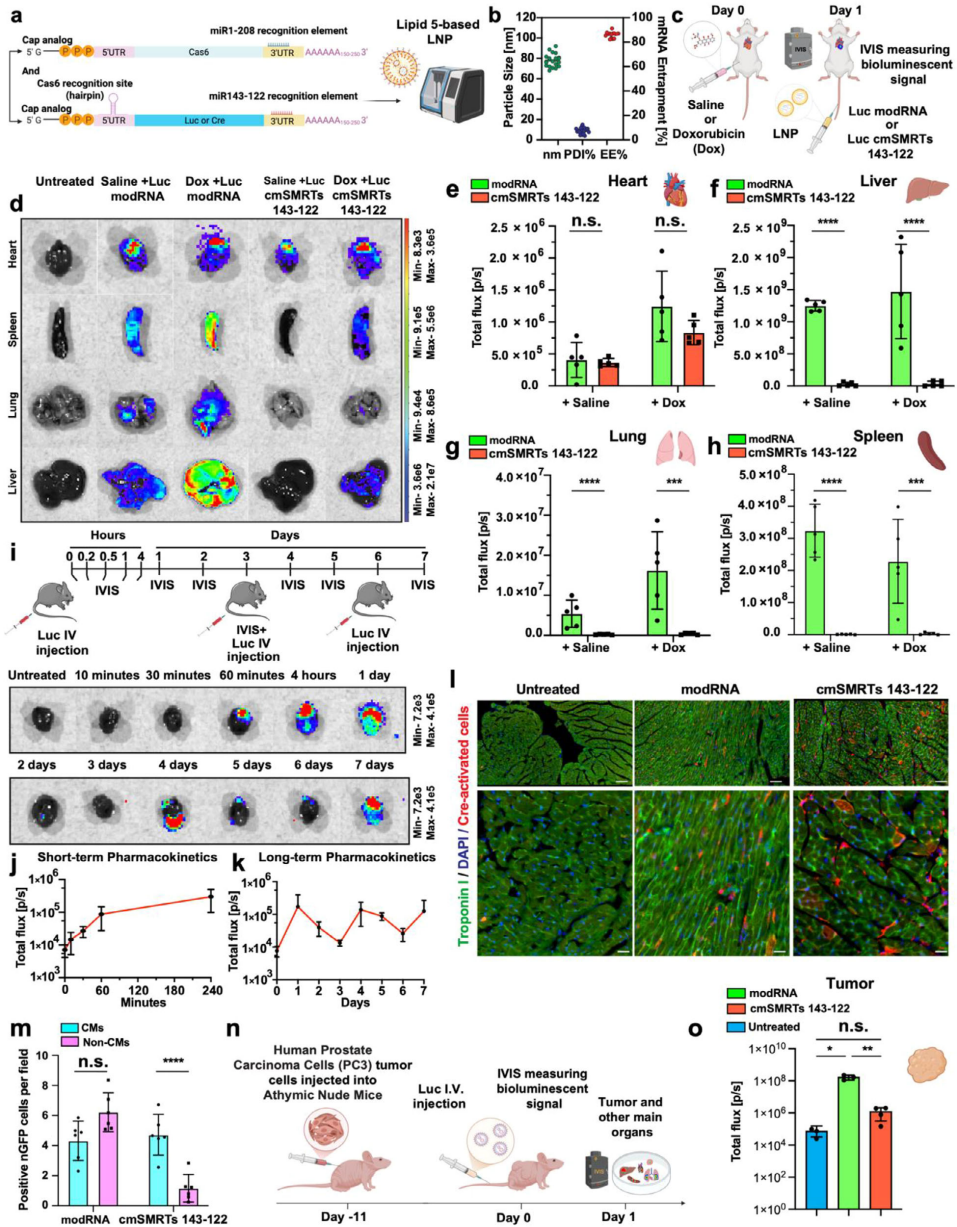
Dual miR‐143/122 recognition elements enhance cardiac selectivity of systemically delivered cmSMRTs. (a) Schematic of the cmSMRTs 143‐122 construct. Cas6 modRNA includes miR‐1 and miR‐208 recognition elements to suppress expression in CMs (CMs), while Luc or Cre modRNA contains Cas6 hairpins and additional miR‐143 and miR‐122 recognition elements to inhibit translation in immune/smooth muscle cells (miR‐143) and hepatocytes (miR‐122). (b) Particle size, polydispersity index (PDI), and RNA entrapment efficiency of LNP‐encapsulated modRNAs, as measured by dynamic light scattering (DLS). (c) Experimental timeline: LNP‐formulated modRNA or cmSMRTs 143‐122 was administered via intravenous (I.V.) injection. (d) Representative IVIS images of Luc expression in major organs 24 h post‐injection. (e–h) Quantification of Luc signal in the heart (e), liver (f), lung (g), and spleen (h). (i) Experimental timeline for pharmacokinetic analysis of LNP‐encapsulated Luc modRNA delivered I.V. (j–k) Short‐term (j) and long‐term (k) Luc expression in the heart over 7 days (*n* = 3). (l) Representative image of Cre expression in cardiac tissue from ROSA26 Cre‐reporter mice following I.V. delivery of Cre cmSMRTs 143‐122. tdTomato+ cells (red) indicate successful Cre recombination. Slides immunostained for CM marker (Troponin I, green) and nuclei (DAPI, blue). The upper panel represents low‐magnification images, while the lower panel represents high‐magnification images. (m) Quantification of Cre‐expressing CMs versus non‐CMs (*n* = 5). (n) Experimental timeline for assessing tumor bioluminescence in human prostate carcinoma (PC3) tumor cells injected subcutaneously into male athymic nude mice after I.V. delivery of Luc modRNA or cmSMRTs 143‐122. (o) Quantification of Luc expression in tumors (*n* = 7–8). Statistical analysis: Two‐way ANOVA was used for (e–h) and (m); one‐way ANOVA with Tukey's multiple comparison test was used for (o); multiple unpaired *t*‐tests were applied for (e–h). Scale bar in l = 100 µm for the upper panel and 30 µm for the lower panel. n.s., not significant; *p < 0.05; **p < 0.01; ***p < 0.001; ****p < 0.0001.

We then characterized the biodistribution and translational selectivity of cmSMRTs 143‐122 (Figure 1a) compared to conventional modRNA. Both formulations showed similar physicochemical properties, with high RNA encapsulation (>90%), ∼80 nm particle size, and low PDI (Figure 1b). Following IV injection, conventional Luc modRNA induced broad expression in all major organs. In contrast, cmSMRTs 143‐122 drove robust cardiac expression with significantly lower signal in non‐cardiac tissues (Figure 1c–h). Quantitative bioluminescence analysis showed approximately 98% less off‐target expression both at baseline and after Dox treatment (Figure S3a,b).

Our next steps were to determine pharmacokinetics and cell specificity in vivo. Pharmacokinetic analysis (Figure 1i–k) showed that cardiac expression of cmSMRTs 143‐122 is abrupt, peaked at 24 h, and declined by day 3. Repeat dosing every three days successfully restored cardiac expression, thus demonstrating that minimally invasive IV injections of cmSMRTs generates controlled, sustained protein production in the heart. To evaluate cell‐type specificity, we delivered Cre‐modRNA via cmSMRTs 143‐122 into ROSA26 reporter mice. Immunofluorescence imaging revealed widespread Cre expression in the heart (marked by red cells), with a significantly higher CM‐to‐non‐CM transfection ratio in cmSMRTs‐treated animals compared to those receiving conventional modRNA (Figure 1l,m).

As Dox can limit the growth of solid tumors, including in prostate and breast cancers [38], expression of AC in tumors could compromise its anti‐cancer efficacy. To explore this, we tested whether cmSMRTs 143‐122, compared to standard modRNA, prevent gene expression in human prostate carcinoma cancer tumors. (Figure 1n). We found that Luc cmSMRTs 143‐122 preserved cardiac expression while eliminating modRNA translation in tumors and other peripheral tissues (Figure 1o and Figure S4). In tumor‐bearing mice receiving Dox treatment, cmSMRTs 143‐122 increased heart‐selective expression 925‐fold and reduced non‐cardiac tissues expression 294‐fold, including in tumors, compared to standard modRNA.

Taken together, these findings establish cmSMRTs 143‐122 as a highly selective, modular, and repeatable systemic gene delivery system capable of directing modRNA translation selectively to the heart and CMs via minimally invasive IV injection. This technology holds significant therapeutic potential for treating heart diseases, including cardiac ischemic injury, while avoiding off‐target effects in other organs as well as tumors.

### 3.2 Acid Ceramidase Preserves Sarcomere Structure, Calcium Handling, and Mitochondrial Function in Cardiomyocytes Exposed to Dox

**FIGURE 2:**
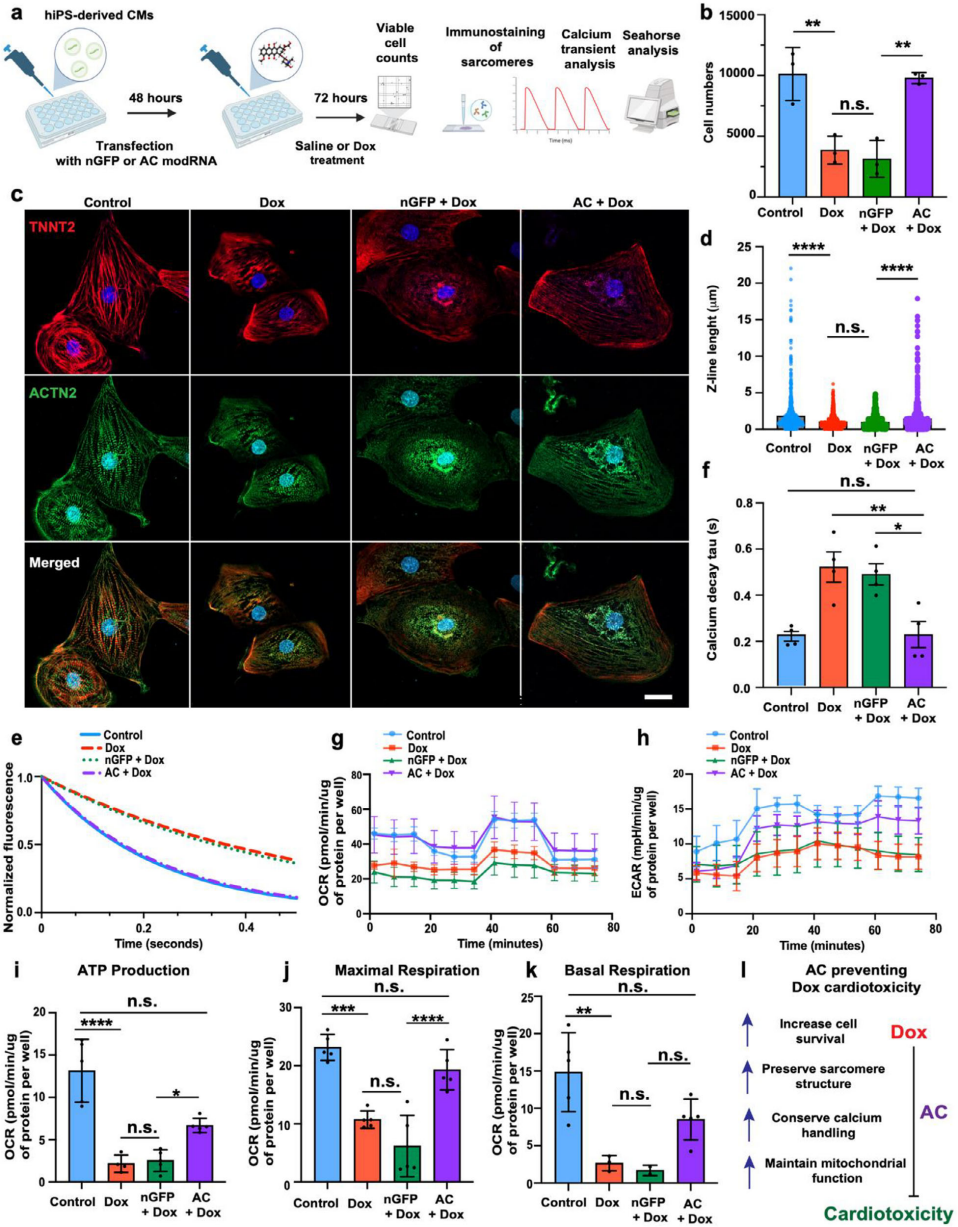
Acid ceramidase (AC) modRNA preserves sarcomere structure, calcium handling, and mitochondrial function in hiPSC‐CMs exposed to Dox. (a) Experimental workflow: hiPSC‐CMs were transfected with AC or control GFP modRNA. After 48 h, cells were treated with 0.5 µm Dox for 72 h before functional and structural assays. (b) Quantification of total nuclei per well, using DAPI staining to assess overall cell viability. (c) Representative immunofluorescence images showing sarcomere structure in untreated control (CTRL), Dox‐treated, and AC+Dox‐treated cells. Cells were stained for cardiac troponin T (TNNT2, red) and sarcomeric α‐actinin (ACTN2, green) (*n* = 3). (d) Quantification of sarcomere length, based on ACTN2 staining (*n* = 700). (e) Representative calcium decay traces measured by Fura‐2 ratiometric imaging in response to electrical stimulation. (f) Quantification of calcium transient decay time (Tau) from Fura‐2 traces (*n* = 3). (g) Seahorse XF analysis showing oxygen consumption rate (OCR) profiles over time during mitochondrial stress testing. (h) Seahorse XF analysis showing oxygen consumption rate (Normalized ECAR) profiles over time during mitochondrial stress testing. (i–k) Quantification of ATP production‐linked respiration (i), maximal respiration after FCCP treatment (j), and basal respiration (k) across groups. (l) the role of AC in preventing Dox cardiotoxicity. Data are presented as mean ± SEM. Statistical analysis: One‐way ANOVA with Tukey's post hoc test was used for group comparisons; sarcomere angle variability was assessed using Levene's test. Scale bar in *c* = 10 µm. n.s., not significant; *p < 0.05; **p < 0.01; ***p < 0.001; ****p < 0.0001.

We previously demonstrated that acid ceramidase (AC) modRNA provides cardioprotection in vivo by modulating ceramide levels and reducing apoptosis following myocardial infraction (MI) [20]. Given the similar molecular mechanisms underlying MI‐ and Dox‐induced cardiac injury, particularly the elevated oxidative stress that leads to ceramide accumulation and apoptosis post injury [7, 11, 17, 24], we hypothesized that AC modRNA may also protects CMs from Dox‐associated cardiotoxicity. To test this hypothesis, we pre‐treated human iPSC‐derived CMs (hiPSC‐CMs) with either AC modRNA or control (nGFP) modRNA. After 48 h, we exposed iPSC‐CMs from both groups to either vehicle or 0.5 µm Dox for an additional 72 h (Figure 2a). Our results show that Dox treatment significantly impedes CM viability, and AC modRNA, but not nGFP modRNA, prevents this decline (Figure 2b). Immunofluorescence staining for cardiac troponin T and alpha actinin 2 (TNNT2 and ACTN2) revealed that Dox exposure disrupted sarcomere structure, shortened sarcomere length, and increased structural disorganization, compared to untreated controls (Figure 2c,d). Notably, pretreatment of iPSC‐CMs with AC modRNA, but not nGFP modRNA, preserved sarcomere integrity (Figure 2c,d). Neonatal mouse CMs had similar outcomes regarding sarcomere structure (Figure S5). Since neonatal CMs exhibit a distinct metabolic profile and are less mature than adult CMs, we focused our functional analyses on hiPSC‐CMs, which more closely model human cardiomyocyte physiology. To further assess functional integrity, we measured calcium transients in hiPSC‐CMs by using Fura‐2‐based ratiometric imaging (Figure S6 and Figure 2f,g). We determined that Dox treatment impaired calcium handling, as reflected by prolonged decay kinetics. Moreover, AC modRNA pretreatment normalized calcium reuptake kinetics, whereas nGFP had no beneficial effect.

Prior studies have shown that Dox inhibits mitochondrial respiration, including in murine iPSC‐CMs [39]. We therefore evaluated the mitochondrial function of hiPSC‐CMs by using Seahorse extracellular flux analysis (Figure 2g–k). We measured the oxygen consumption rate (OCR, Figure 2g), which indicates mitochondrial respiration, and the extracellular acidification rate (ECAR, Figure 2h), which quantifies glycolysis, in hiPSC‐CMs in real time. We observed that Dox exposure lowers both OCR and ECAR in hiPSC‐CMs (Figure 2g,h). However, pre‐treatment with AC but not nGFP modRNA prevented this drop in cell metabolism. Dox also significantly decreased ATP‐linked respiration (Figure 2i), maximal respiratory capacity (Figure 2j), and basal respiration (Figure 2k) in a hiPSC‐CM in vitro model. These deficits were attenuated in cells pretreated with AC modRNA but not nGFP modRNA, thus indicating that AC modRNA pretreatment preserved cell survival, sarcomere structure, calcium handling, and mitochondrial bioenergetics (Figure 2l).

To explore the in vivo relevance of ceramide in Dox‐induced cardiotoxicity, we administered a single dose of Dox (5 mg/kg) to adult male C57BL/6 mice and harvested hearts one day later for transcriptomic analysis. We chose a one‐day post‐Dox timepoint to capture early transcriptional changes associated with ceramide biosynthesis and apoptosis. This timing aligns with prior studies identifying early transcriptomic shifts that precede structural changes [20]. RNA sequencing demonstrated upregulation of pro‐ceramide biosynthesis and pro‐apoptotic genes, along with downregulation of anti‐apoptotic transcripts in the myocardium (Figure S7a,b). These findings were confirmed by qPCR (Figure S7c).

Finally, we tested whether systemic delivery of encapsulated AC or Luc via cmSMRTs 143‐122 could selectively elevate AC in the heart and reduce Dox‐associated ceramide accumulation and apoptosis (Figure S8a). For this, mice were pretreated with a single IV injection of encapsulated Luc or AC cmSMRTs 143‐122 one day prior to Dox administration. Control groups were Dox‐treated or untreated mice. We observed that Dox administration resulted in higher ceramide accumulation in the heart and that AC, but not Luc, treatment significantly lowered ceramide levels in the heart (Figure S8b). Further, we found that AC expression was upregulated in the heart (Figure S8c) but not in other organs such as the lung, spleen, or liver (Figure S8d–f). Finally, we showed that, compared to Luc, AC significantly decreased Caspase‐3 transcript levels in the heart 24 h post‐Dox treatment, results that suggest attenuated Dox‐induced apoptosis (Figure S8g).

Overall, these findings provide insight into the therapeutic impact and support the cardioprotective role of AC cmSMRTs 143‐122 in the context of Dox exposure, specifically by demonstrating that IV delivery of encapsulated AC cmSMRTs 143‐122 selectively elevated AC expression in the heart and not in other vital organs, reduced ceramide accumulation in the heart, and mitigated apoptosis post Dox treatment in vivo (Figure 2l).

### 3.3 AC cmSMRTs 143‐122 Prevents Cardiac Dysfunction, Fibrosis, and Atrophy in a Chronic Dox‐Induced Cardiotoxicity Model

**FIGURE 3:**
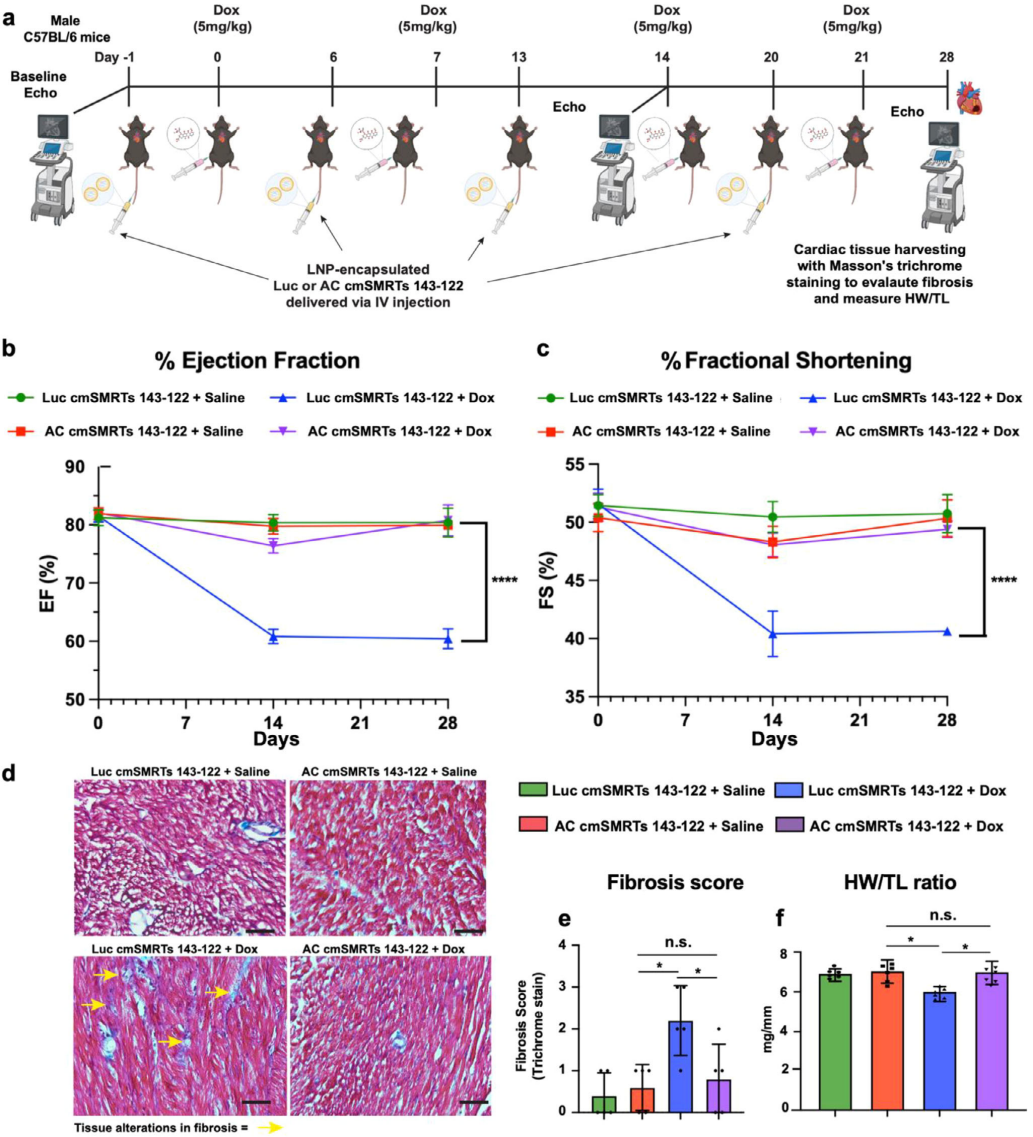
Pretreatment with AC cmSMRTs 143‐122 prevents Dox‐associated cardiotoxicity in a male C57BL/6 mouse model. (a) Experimental timeline: Mice were pretreated with LNP‐encapsulated Luc or AC cmSMRTs 143‐122 via intravenous (IV) injection on days ‐1, 6, 13, and 20. One day after each pretreatment, mice received intraperitoneal (IP) injections of saline or Dox (5 mg/kg). Echocardiography was performed on days −1, 14, and 28 to assess cardiac function. On day 28, hearts, tibias, and blood were collected for evaluations of cardiac atrophy, fibrosis, hematologic parameters, and liver enzyme levels (*n* = 6). (b,c) Echocardiographic quantification of left ventricular function: % ejection fraction (b) and % fractional shortening (c) over the course of treatment. (d) Representative Masson's trichrome staining of cardiac tissue. Yellow arrows indicate regions of fibrotic remodeling. (e) Quantification of fibrosis score after different treatments (*n* = 5). (f) Quantification of heart‐weight‐to‐tibia‐length (HW/TL) ratio as a measure of cardiac atrophy on day 28 (*n* = 6). Statistical analysis: Two‐way ANOVA was used for (b,c); one‐way ANOVA with Tukey's multiple comparison test was used for (e,f) Scale bar in *d *= 50 µm. n.s., not significant; *p < 0.05; ****p < 0.0001.

To assess the cardioprotective efficacy of AC cmSMRTs 143‐122 in vivo, we employed a clinically relevant chronic model of Dox‐induced cardiotoxicity (Figure 3). Adult male C57BL/6 mice were pretreated with LNP‐encapsulated Luc (control) or AC cmSMRTs 143‐122 via IV injection. One day later, animals received intraperitoneal Dox (5 mg/kg) or saline. This cycle was repeated weekly for four weeks. Cardiac function was monitored using echocardiography at baseline, day 14, and day 28. Hearts were then harvested for histological analyses (Figure 3a). As expected, mice pretreated with Luc cmSMRTs + Dox exhibited significant declines in both % ejection fraction (%EF) and % fractional shortening (%FS) at days 14 and 28 post‐treatment, changes that indicate impaired systolic function (Figure 3b,c). In contrast, pretreatment with AC cmSMRTs 143‐122 before Dox administration preserved cardiac contractility, in that %EF and %FS levels remained comparable to those of saline‐treated controls. Histological assessment using Masson's trichrome staining displayed altered myocardial fibrosis in the Luc cmSMRTs 143‐122 + Dox group, while hearts from AC cmSMRTs 143‐122 + Dox‐treated mice showed minimal fibrotic remodeling and lower fibrosis scores (Figure 3d,e). Moreover, Dox treatment led to, as expected, cardiac atrophy in mice pretreated with Luc but not AC cmSMARTs 143‐122 (Figure 3f).

To determine whether repeated systemic administration of cmSMRTs 143‐122 causes off‐target toxicity, we conducted a comprehensive safety analysis (Figure S9a). Histological examination of the heart, liver, lungs, and spleen revealed no evidence of tissue damage or abnormalities (Figure S9b). Complete blood counts, including of lymphocytes, neutrophils, monocytes, eosinophils, and platelets, showed no significant alterations across treatment groups (Figure S9c–g). In addition, serum liver enzymes, ALP, AST, and ALT, remained within normal ranges, thus indicating no hepatic dysfunction despite repeated LNP dosing (Figure S9h–j).

Together, these findings demonstrate that systemic administration of AC cmSMRTs 143‐122 effectively prevented Dox‐induced cardiac dysfunction, fibrosis, and atrophy, without eliciting systemic toxicity. Further, our results highlight the potential AC cmSMRTs 143‐122 has as a clinically translatable strategy for protecting the heart during anthracycline chemotherapy.

### 3.4 AC cmSMRTs 143‐122 Preserves Cardiac Function without Compromising the Anti‐Tumor Activity of Doxorubicin in a PC3 Human Prostate Tumor Model

Because Dox is typically used to prevent tumor growth [38] as part of cancer treatment, we wanted to ensure that the cardioprotective effects of AC cmSMRTs 143‐122 do not interfere with Dox's anti‐tumor efficacy. To explore this, we evaluated AC cmSMRTs 143‐122's cardioprotective impact in a chronic Dox cardiotoxicity model in male athymic nude mice bearing human prostate carcinoma (PC3) tumors. Five groups of mice were inoculated with PC3 tumor cells; four of these groups were treated with Dox (5 mg/kg), and one group was left untreated. One Dox‐treated group received no modRNA injections (Dox), while the other three groups received IV LNP‐encapsulated AC cmSMRTs 143‐122, AC modRNA, or Luc cmSMRTs 143‐122. This cycle was repeated weekly over a 28‐day period.

**FIGURE 4:**
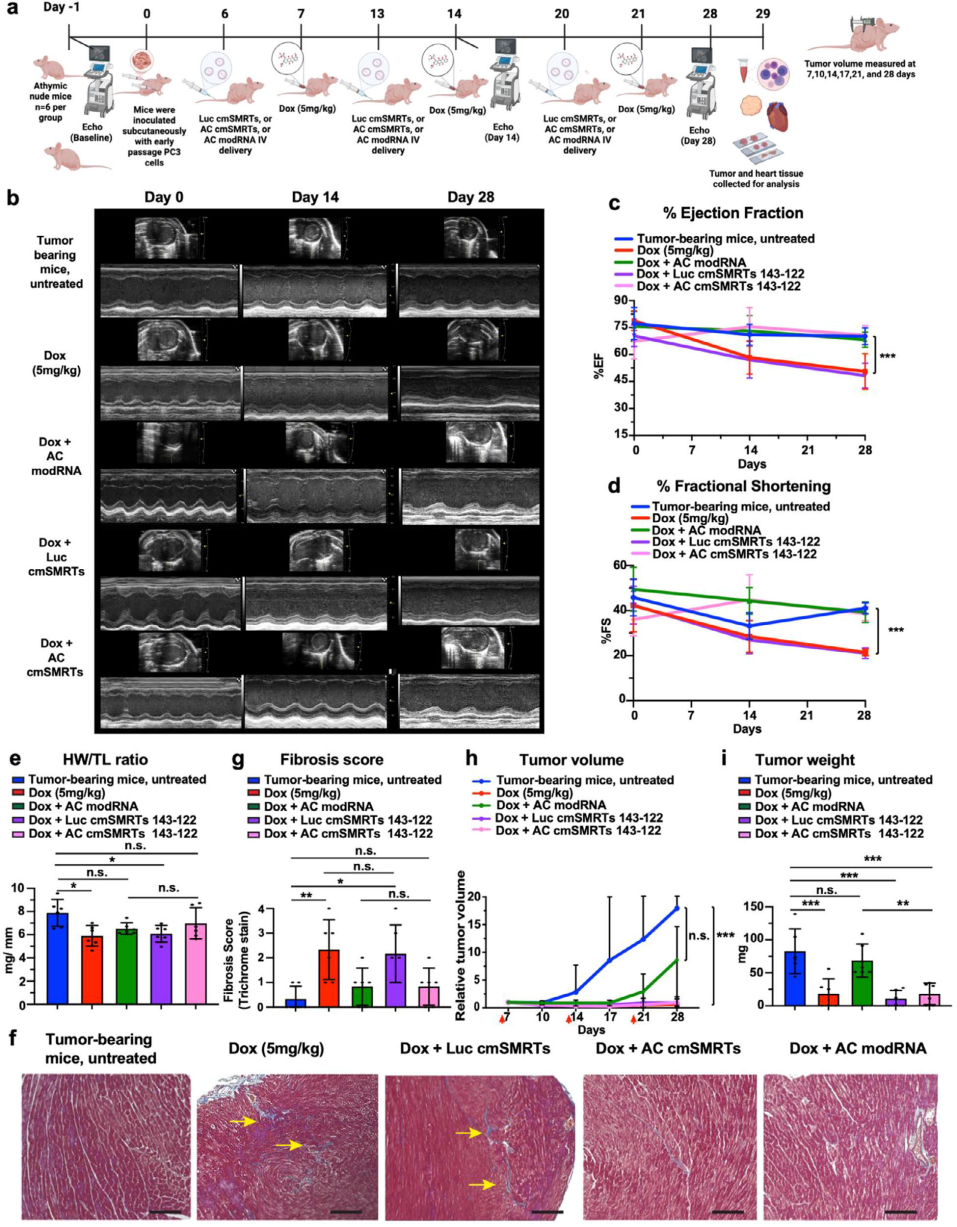
Pretreatment with AC cmSMRTs 143‐122 prevents Dox‐induced cardiotoxicity without compromising anti‐tumor efficacy in male athymic nude mice bearing human prostate carcinoma cells (PC3) tumors. (a) Experimental timeline: Tumor‐bearing male athymic nude mice (*n* = 6) were allocated to five groups. One group remained untreated (Tumor‐bearing mice, untreated). The second group received Dox treatment (5 mg/kg) weekly at days 7, 14, and 21. The other three groups were pretreated with IV injections of LNP‐encapsulated Luc cmSMRTs 143‐122 (Dox + Luc cmSMRTs 143‐122), AC modRNA (Dox + AC modRNA), or cmSMRTs 143‐122 (Dox + Ac cmSMRTs 143‐122) on days 6, 13, and 20. One day after each treatment dose, mice received IP injections of Dox (5 mg/kg). Echocardiography was performed on days −1, 14, and 28. On day 29, hearts, tumors, tibias, and blood were collected to assess cardiac atrophy, complete blood count, and liver enzyme levels. (b) Representative M‐mode echocardiographic images from all treatment groups at multiple time points c and d, Echocardiographic assessment of cardiac function showing % ejection fraction (c) and % fractional shortening (d) at the indicated time points. (e) Quantification of heart‐weight‐to tibia‐length (HW/TL) ratio to assess cardiac atrophy at experimental endpoint (day 29). (f) Relative tumor volume measurements over time for each treatment group. (g) Tumor weight (mg) at experimental endpoint (day 29). (h) Representative Masson's trichrome staining of cardiac tissue to determine cardiac fibrosis at experimental endpoint (day 29). Yellow arrows indicate regions of fibrotic remodeling. (i) Quantification of fibrosis score after different treatments (*n* = 6). Statistical analysis: Two‐way ANOVA was used for (c,d,f). One‐way ANOVA with Tukey's multiple comparison test was used for (e,g,i). Scale bar in *h* = 50 µm. Statistical analysis: n.s., not significant; *p < 0.05; **p < 0.01; ***p < 0.001.

Tumor growth, cardiac function, and organ‐specific toxicity were monitored throughout the study (Figure 4a). Cardiac function was assessed through echocardiography (echo). Figure 4b shows a representative M‐mode echo for different groups on days 0, 14, and 28. Cardiac analysis revealed that mice pretreated with either Dox only (i.e., without modRNA) or Luc cmSMRTs 143‐122 followed by Dox exhibited significant cardiac dysfunction, as characterized by reduced %EF (Figure 4c) and %FS (Figure 4d). In contrast, mice pretreated with either AC cmSMRTs 143‐122 or AC modRNA maintained cardiac contractility at levels similar to those of untreated controls (Figure 4b–d). These results using athymic nude mice mirrored findings in non‐tumor‐bearing C57BL/6 mice and thus confirmed that AC cmSMRTs 143‐122 is protective across strains and disease models. We also measured heart‐weight‐to‐tibia‐length (HW/TL) ratio to determine cardiac atrophy and found that Dox‐treated mice receiving either Dox only or Dox plus Luc cmSMRTs 143‐122 had significantly decreased HW/TL, while pretreatment with either AC cmSMRTs 143‐122 or modRNA preserved normal heart mass (Figure 4e). Moreover, histological quantification using Masson's trichrome staining indicated more fibrosis (Figure 4f,g) in the hearts of mice that were treated with either Dox only or Dox plus Luc cmSMRTs 143‐122, while hearts from mice treated with either AC cmSMRTs 143‐122 or AC modRNA showed minimal fibrotic remodeling and lower fibrosis scores (Figure 4f,g). Importantly, tumor volume over time (Figure 4h) and endpoint tumor weight (Figure 4i) were significantly diminished in Dox‐treated mice that received either AC or nGFP cmSMRTs 143‐122 but not those that received AC modRNA, compared to mice that were not administered Dox (Figure 4h,i). These findings show that only AC cmSMRTs 143‐122, and not AC modRNA, facilitate selective AC expression in the heart but less expression in the tumor, thereby producing cardioprotective effects without compromising Dox's oncologic efficacy against human PC3 tumors (Figure 4). These results validate both the cardiac selectivity of the cmSMRTs 143‐122 platform and its translational safety in the context of combination cancer therapy. Consistent with findings in non‐tumor‐bearing mice models, additional safety evaluations, including histology of the liver and spleen, blood cell profiling, and liver enzyme measurements (Figure S10), demonstrated no pathological changes in response to cmSMRTs miR‐143‐122 treatment, nor hematological or hepatic toxicity from repeated LNP or modRNA exposure (Figure S10).

Together, these results establish that AC cmSMRTs 143‐122 effectively protect the heart from anthracycline‐induced toxicity without diminishing Dox's therapeutic efficacy against human prostate cancer.

### 3.5 AC cmSMRTs 143‐122 Preserves Cardiac Function without Compromising the Anti‐Tumor Activity of Doxorubicin in a Female 4T1 Breast Tumor Model

Our previous mouse models (athymic nude and C57BL/6 mice) used only male mice. Athymic nude mice are also immunocompromised. Athymic nude mice lack a functional thymus gland, which impairs its immune system by preventing the development of mature T‐cells. Therefore, to expand the scope of our inquiry, we evaluated AC cmSMRTs 143‐122's cardioprotective effects in 4T1 breast tumor‐bearing female immunocompetent Balb/c mice. First, we established that cmSMRTs 143‐122 conserved cardiac expression while significantly limiting modRNA translation in tumors and other peripheral tissues in this model (Figure S11). In tumor‐bearing mice receiving Dox treatment, cmSMRTs 143‐122 increased heart‐specific expression 185‐fold and decreased expression 246‐fold in non‐cardiac tissues, including tumors, compared to standard modRNA.

Next, Balb/c mice were inoculated with 4T1 tumor cells and then systemically pretreated with either LNP‐encapsulated AC or control nGFP cmSMRTs 143‐122. One day after pretreatment, animals received either Dox (5 mg/kg) or saline. This cycle was repeated weekly over a 28‐day period. Tumor growth, cardiac function, and organ‐specific toxicity were monitored throughout the study (Figures S12,S13). Similar to our results in athymic nude and C57BL/6 mice, in 4T1 tumor‐bearing Balb/c mice, cardiac analysis indicated that nGFP cmSMRTs 143‐122 plus Dox treatment led to reduced %EF (Figure S12b) and %FS (Figure S12c), while either nGFP cmSMRTs 143‐122 + Saline or AC cmSMRTs 143‐122 + Dox preserved cardiac contractility. Hearts weight was significantly lesser post nGFP cmSMRTs 143‐122 + Dox treatment compared to either nGFP cmSMRTs143‐122 + saline or to AC cmSMRTs 143‐122 + Dox (Figure S12d). Importantly, tumor volume over time (Figure S12e) and endpoint tumor weight (Figure S12f) were significantly diminished in both the AC and nGFP cmSMRTs 143‐122 + Dox groups, compared to the nGFP cmSMRTs143‐122 + saline group. Safety assessments, including liver enzyme measurements and blood cell profiling, showed minimal hepatic response to LNP treatment and fewer lymphocytes due to Dox treatment. These results confirm that AC cmSMRTs 143‐122 have cardiac selectivity in female Balb/c mice and avoid translation in 4T1 breast cancer tumors, thus enabling cardiac protection without compromising Dox's anti‐tumor activity and without causing any severe safety concerns.

Overall, the results presented here demonstrate that IV delivery of AC cmSMRTs 143‐122 safely protects the heart against Dox cardiotoxicity without compromising its ability to limit tumor growth. By studying male C57BL/6 and PC3 prostate tumor‐bearing athymic nude mice as well as female 4T1 breast tumor‐bearing Balb/c mice, we have confirmed that AC cmSMRTs 143‐122 exerts these benefits across strains, sexes, and disease models. Moreover, these findings emphasize the clinical potential of this platform for effective cardio‐oncology applications.

## 4 Discussion

Chemotherapy remains a cornerstone of cancer treatment; however, its use is often limited by off‐target toxicities. Among these side effects, cardiotoxicity is one of the most clinically significant and well‐documented, especially with anthracycline drugs such as Dox [40]. Though it successfully suppresses tumor growth, Dox deleteriously impacts the heart by both inducing oxidative stress and promoting in cardiac cells the accumulation of ceramides, which are lipid signaling molecules that trigger apoptosis and compromise myocardial function [41].

We previously demonstrated that AC modRNA, when delivered directly into the heart post‐MI, reduced ceramide accumulation, limited cell death and inflammatory cell infiltration, and preserved cardiac function [20]. We also developed and validated a modRNA circuit, cmSMRTs, that allows selective expression of modRNA in CMs [26, 27]. Building on these findings, the present study introduces a novel, minimally invasive gene therapy platform that combines LNP‐encapsulated CM‐selective modRNA expression with systemic IV delivery to protect the heart from Dox‐induced cardiotoxicity.

Unlike traditional cardiac gene therapy methods requiring open‐chest surgery and intramyocardial injections, our cmSMRTs platform enables IV‐administered modRNA to be translated selectively in the heart, while minimizing off‐target expression in other tissues—including tumors (Figure 1 and Figures S1–S4,S11). We here show that delivering AC to hiPSC‐CM preserves sarcomere structure, calcium handling, and mitochondrial function in vitro (Figure 2 and Figures S5,S6). In vivo, we used a murine short‐term Dox exposure model and found that while Dox induced ceramide accumulation in the heart, pretreatment with cardiac‐selective AC cmSMRTs 143‐122 attenuated this accumulation and also mitigated Caspase‐3, a marker for apoptosis (Figures S7,S8). Additionally, we explored long‐term Dox exposure in three different murine models: male C57BL/5 mice, male athymic nude mice bearing human prostate carcinoma cells (PC3) tumors, and female Balb/c mice bearing breast cancer tumors (4T1). These in vivo experiments determined that AC cmSMRTs 143‐122 prevented the atrophy, cardiac fibrosis, and contractility dysfunction associated with Dox treatment without compromising its oncologic efficacy against prostate or breast tumors (Figures 3, 4 and Figure S12). We also demonstrate the safety and tolerability of our IV delivery treatment: in all three models, repeated dosing of AC cmSMRTs 143‐122 did not damage liver function or lead to histological abnormalities or hematological dysfunction (Figures S9,S10,S13). Importantly, while encapsulated AC modRNA can induce cardioprotection when delivered IV, it also compromises Dox's anti‐tumor activity. This emphasizes the need to selectively deliver AC to the heart without generating AC expression in the tumor, a balance that only AC cmSMRTs 143‐122 was able to achieve.

Seeking to mitigate Dox cardiotoxicity, recent studies have explored alternative approaches, including small molecule inhibitors [30] and liposomal drug carriers [42]. However, these techniques lack tissue‐cell‐type selectivity and do not offer a modular, programmable platform for broader cardiac gene therapy. Our method addresses this gap by adding tissue‐enriched miR recognition elements in order to enable a gene‐of‐interest to be selectively translated in the heart. This development makes our system adaptable for other therapeutic targets and cardiac conditions (e.g., to treat ischemic heart disease).

One limitation of our current protocol is its use of LNPs based on Lipid 5, which lacks cardiac tropism and predominantly favors liver uptake. Although our IV‐delivered cmSMRTs effectively suppress expression in non‐cardiac tissues, optimizing LNP composition, by either using more cardiotropic lipids or incorporating targeting moieties such as cardiac‐specific peptides or antibodies, could further enhance delivery efficiency and reduce residual off‐target translation. Importantly, changes in LNP formulation may alter surface charge and biodistribution profiles [43], thereby necessitating re‐optimization of miR recognition elements for each lipid formulation.

As expected, intramyocardial delivery produces significantly higher cardiac translation compared to IV delivery (∼90% [23, 25] vs. 2.3%, 3.7%, or 18.5% in different models). That said, by avoiding the risks of open chest surgery, such as bleeding, infection, and arrhythmias [44], our platform offers major clinical advantages over intramyocardial methods. Interestingly, we observed (Figures S3,S4,S11) that Luc cmSMRTs 143‐122 have dramatically different cardiac selectivity in C57BL/6 mice (2.3%), Balb/c (3.7%), and athymic nude mice (18.5%), probably due to skin and hair color. These differences show that using IVIS to measure translation in vivo carries technical limitations. A more quantitative method is needed to evaluate the cardiac selectivity of encapsulated Luc cmSMRTs 143‐122. Additionally, our cmSMRTs 143‐122 produce significantly lower expression than modRNA. Because Luc cmSMRTs 143‐122 significantly repressed Luc translation in organs other than the heart, the observable Luc signal after IV delivery of Luc cmSMRTs 143‐122 represents only a small percentage of the total expression achieved by IV‐injected modRNA (Figures S3,S4,S11). Utilizing cardiotropic LNPs may balance this equation and allow a higher percentage of Luc to be translated.

Our findings not only position AC cmSMRTs 143‐122 as a promising therapeutic to counter anthracycline cardiotoxicity but also establish the cmSMRTs 143‐122 platform as a versatile delivery platform for precision cardiac gene therapy. Indeed, our approach provides a scalable, repeatable, and programmable system for evaluating therapeutic genes in various diseases inside and outside the cardiac field (e.g., cancer SMRTs).

A critical next step will be to assess the translatability of this approach in large animal models, such as pigs, using both IV and catheter‐based intracoronary delivery. Success in this setting would pave the way for future large animal pre‐clinical and clinical trials and may ultimately lead to selective cardioprotective treatment for cancer patients being treated with Dox. In the broader aspect, our minimally invasive platform could eliminate the need for open chest surgery and intramyocardial injection for cardiac gene delivery. In summary, by combining molecular precision with practical translational potential, our CM‐selective modRNA delivery strategy represents a new direction in cardio‐oncology and, more broadly, cardiac‐selective mRNA therapy.

## Author Contributions

J.Y. and G.M. designed and performed most of the experiments, analyzed the majority of the data, and co‐wrote the manuscript. They contributed equally to this work. M.G. conducted experiments evaluating AC modRNA function in hiPSC‐CMs treated with Dox. B.G.D.S. and N.B. performed Seahorse metabolic analyses. M.M.Z., K.K., A.A.K., M.A., S.S., E.M., L.Z., D.H., S.P., R.H., and J.L.E.A. contributed to modRNA preparation, histology, immunostaining, qPCR, echocardiography; established the PC3, 4T1 tumor‐bearing athymic nude, and Balb/c mouse models; and revised the manuscript. N.D. provided the hiPSC‐CMs. L.G. supervised the Seahorse experiments. M.S. edited and revised the manuscript. L.Z. conceived and designed the study, analyzed data, and co‐wrote the manuscript.

## Conflicts of Interest

L.Z., K.K., and M.M.Ż. are listed as inventors on a PCT application, entitled *Regulatory System for Expression of a Gene of Interest in a Target Cell and Methods of Use Thereof* (PCT/US002/05349), which covers findings reported in this manuscript.

## Supporting information




**Supporting File**: advs73452‐sup‐0001‐SuppMat.docx.

## Data Availability

The data that support the findings of this study are available from the corresponding author upon reasonable request.
