# Supplementary material for: A Systemic Selective Modified mRNA Delivery Platform for Preventing Chemotherapy‐Induced Cardiotoxicity
Source: Adv Sci (Weinh). 2026 Jan 16;13(10):e10543. doi: 10.1002/advs.202510543 (PMC12915083; doi:10.1002/advs.202510543)
Supplement: Supplementary file 1 — Supporting File: advs73452‐sup‐0001‐SuppMat.docx. [file ADVS-13-e10543-s001.docx]

**Supplementary data**

**A systemic selective modified mRNA delivery platform for preventing chemotherapy-induced cardiotoxicity**

Jimeen Yoo^*^, Gayatri Mainkar^*^, et.al.

Corresponding author: Email: lior.zangi@mssm.edu.

* These authors contributed equally to this work.

This PDF file includes:

1. Supplemental Figures 1-13
2. Supplemental Tables 1-3

**Supplemental Figures**

**
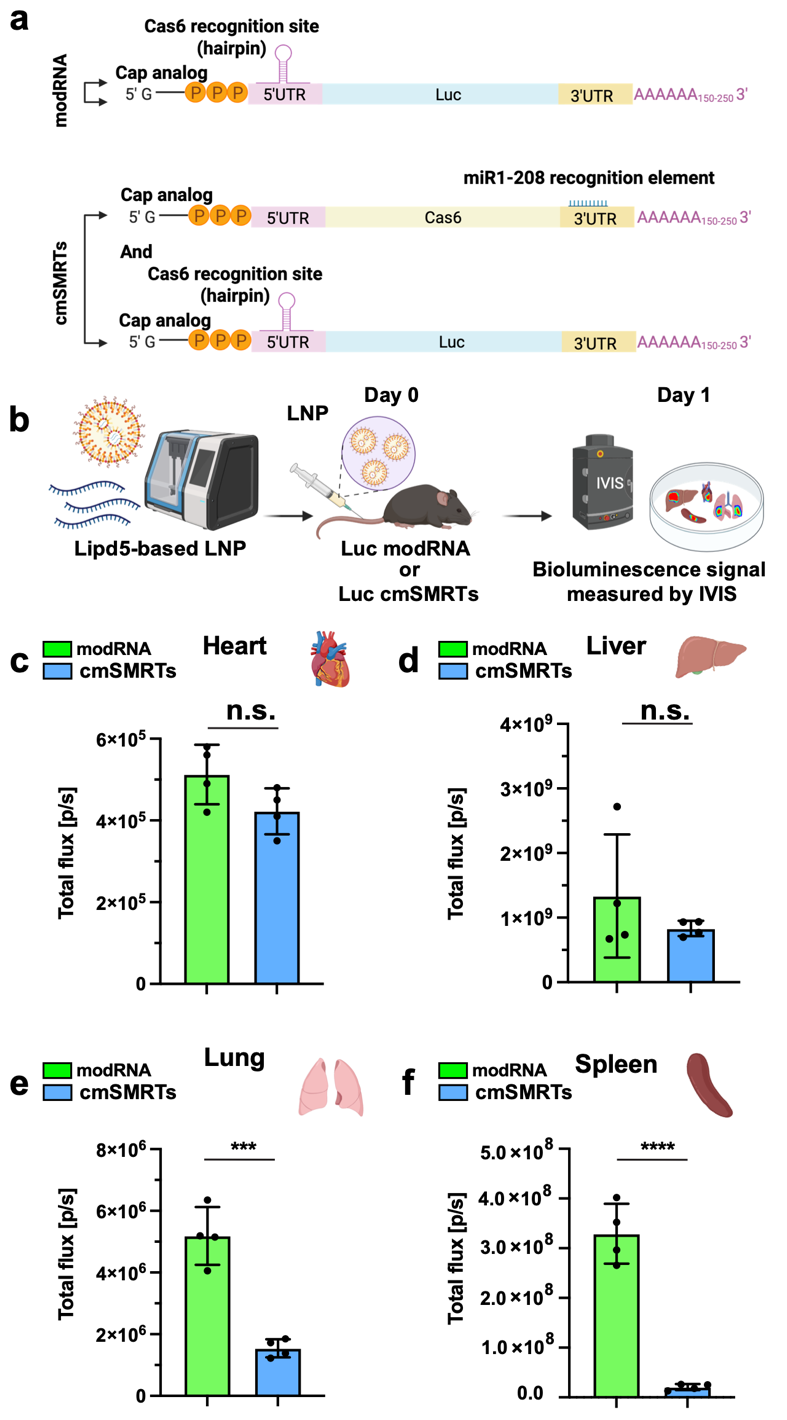
**

**Supplementary Figure 1. IV delivery of cmSMRTs significantly reduces modRNA translation in the lung and spleen while maintaining expression in the heart and liver.** **a**, Schematic of the modRNA and cmSMRTs constructs. Cas6 modRNA includes miR-1 and miR-208 recognition elements to suppress translation in CMs. Luc modRNA contains Cas6 recognition hairpins to block expression in non-target tissues. **b**, Experimental timeline: LNP-formulated Luc modRNA or cmSMRTs was administered via IV injection. Twenty-four hours post-injection, bioluminescence imaging (IVIS) was used to assess Luc expression in major organs. **c–f**, Quantification of Luc expression in the heart (**c**), liver (**d**), lung (**e**), and spleen (**f**). Statistical analysis: Unpaired t-test was used for **c–f**. n.s., not significant; ***p < 0.001; ****p < 0.0001.

**
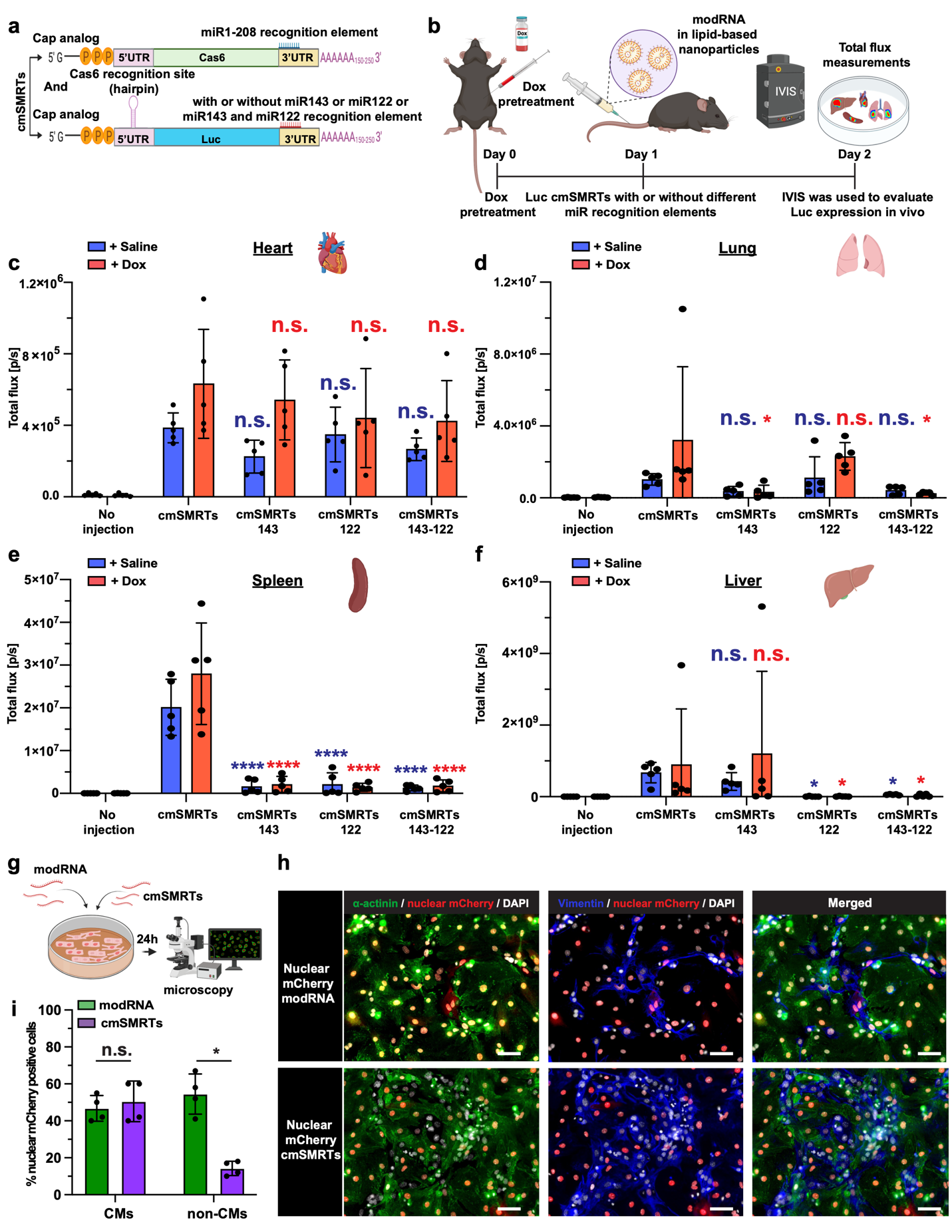
**

**Supplementary Figure 2. miRNA recognition elements reduce off target modRNA translation in non-cardiac tissues following IV delivery.** **a**, Structural representation of cmSMRTs constructs: Cas6 modRNA contains miR-1 and miR-208 (miR1–208) recognition elements to restrict translation in CMs. Luc modRNA includes Cas6 recognition hairpins and additional miR recognition elements—miR-143 (smooth muscle), miR-122 (liver), or both (miR143-122)—to suppress translation in non-target tissues. **b**, Experimental timeline: Mice received systemic intravenous (IV) injections of LNP-formulated Luc modRNA variants (cmSMRTs, cmSMRTs-143, cmSMRTs-122, or cmSMRTs-143-122). Prior to IV delivery, mice were injected intraperitoneally (IP) with either saline or Dox (5 mg/kg). Twenty-four hours later, bioluminescence imaging was used to assess tissue-specific Luc expression. **c–f**, Quantification of Luc expression in the heart (**c**), lung (**d**), spleen (**e**), and liver (**f**) 24 hours post-injection (n = 5). **g**, Experimental timeline: in vitro neonatal cardiac cultures transfected for 24 hours with nuclear mCherry modRNA or cmSMRTs constructs. Cells were fixed and stained for mCherry expression (red), α-actinin (CM marker, green), vimentin (mom-CM marker, blue), and DAPI (nuclei, white) followed by fluorescence microscopy. **h**, Representative immunofluorescence images of neonatal cardiac cultures transfected with nuclear mCherry modRNA or cmSMRTs. **i**, Quantification of % positive transfection to CMs or non-CMs post transfection with nuclear mCherry modRNA or cmSMRTs. Statistical analysis: Two-way ANOVA for c–f and multiple unpaired t tests for i. n.s., not significant; *p < 0.05; ****p < 0.0001. Scale bar in **h** = 50μm.

**
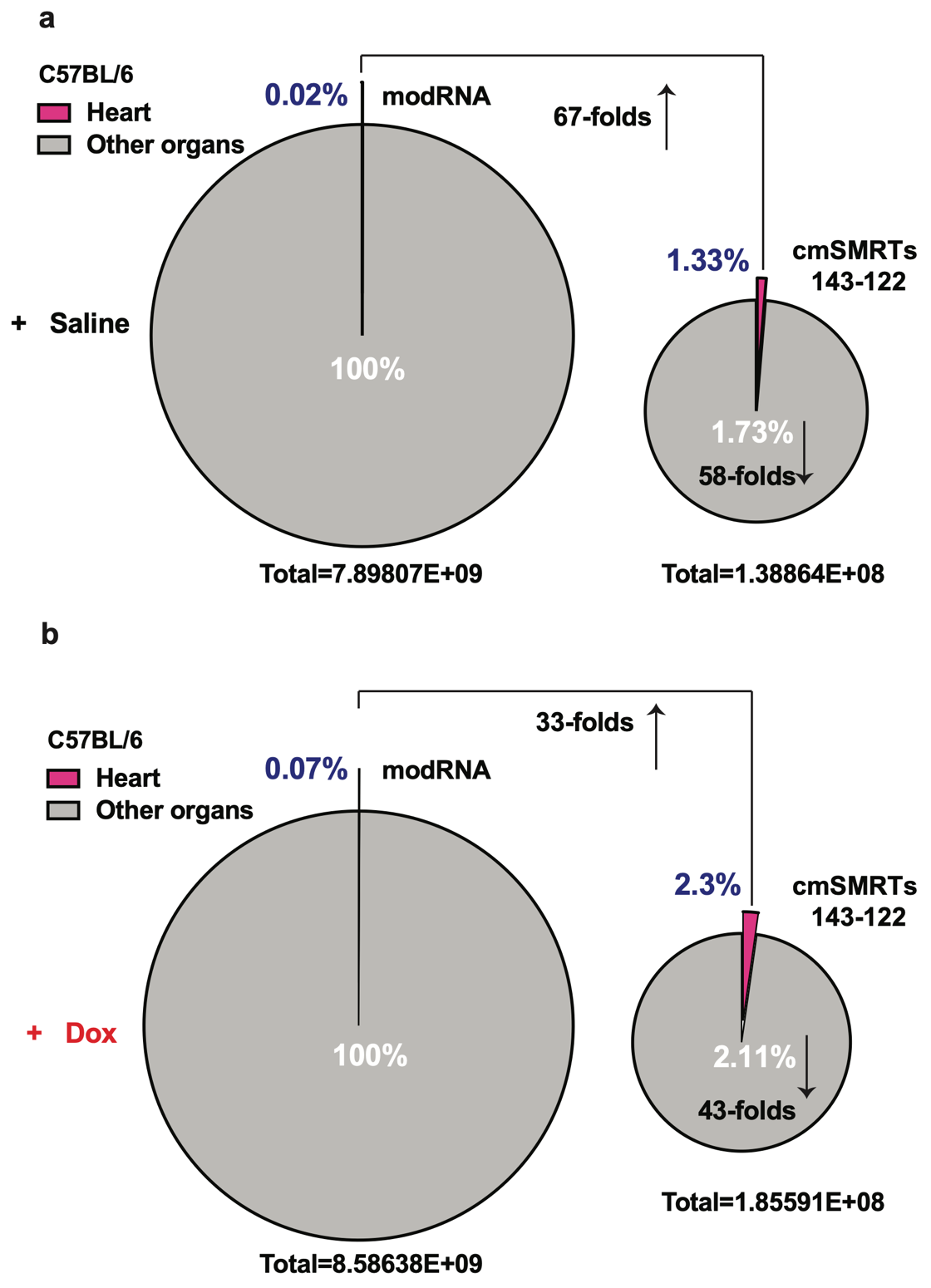
**

**Supplementary Figure 3. Cardiac selectivity of cmSMRTs 143-122 following IV delivery in C57BL/6 mice with or without Dox treatment.** **a–b**, Pie chart representations of overall bioluminescence signal distribution following IV delivery of Luc modRNA or cmSMRTs 143-122 in C57BL/6 mice. Charts display percentages of total expression localized in the heart compared to other organs under baseline, (**a**) saline-treated, and (**b**) Dox-treated conditions. Percentages in blue indicate proportions of total expression localized in the heart. Percentages in white represent cardiac expression relative to conventional Luc modRNA. Total luminescence signal for each condition is noted beneath the corresponding pie chart.

**
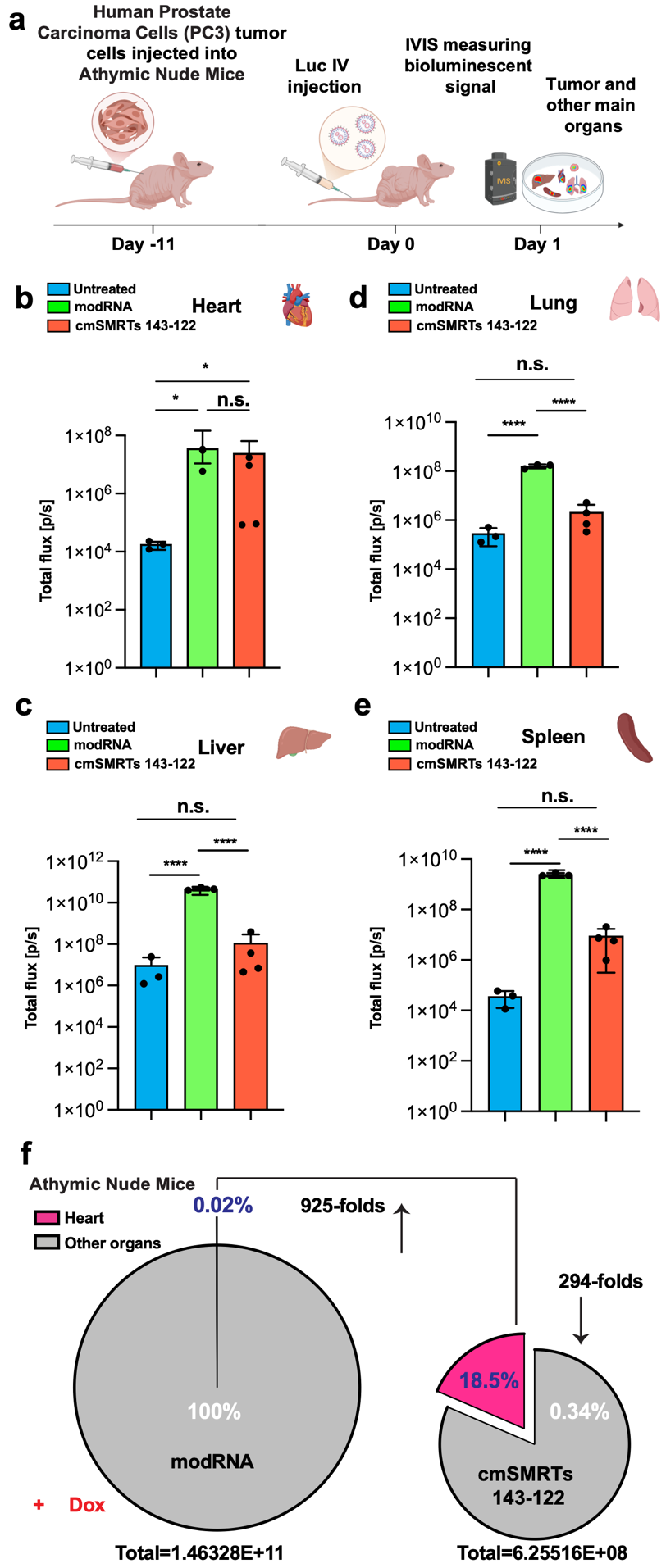
**

**Supplementary Figure 4. Tissue-specific expression following IV delivery of Luc modRNA or cmSMRTs 143-122 in human prostate carcinoma cells (PC3) tumor-bearing athymic nude mice.** **a**, Experimental timeline: athymic nude mice bearing PC3 breast tumors received IV injections of LNP-formulated Luc modRNA or cmSMRTs 143-122. Bioluminescence imaging (IVIS) was performed 24 hours post-injection to assess Luc expression in tumors and major organs. **b–e**, Quantification of Luc signal in the heart (**b**), liver (**c**), lung (**d**), and spleen (**e**) after IV delivery (n = 4) or without treatment (n=3). **f**, Pie chart comparing the distribution of total bioluminescence signal following IV delivery of Luc modRNA or cmSMRTs 143-122 in Dox-treated mice. Percentages in blue indicate the proportion of total expression localized in the heart. Percentages in white represent heart-specific expression relative to conventional Luc modRNA. Total luminescence values for each condition are listed beneath each chart. Statistical analysis: One-way ANOVA with Tukey’s multiple comparison test was used for **b–e**. n.s., not significant; ***p < 0.001; ****p < 0.0001.

**
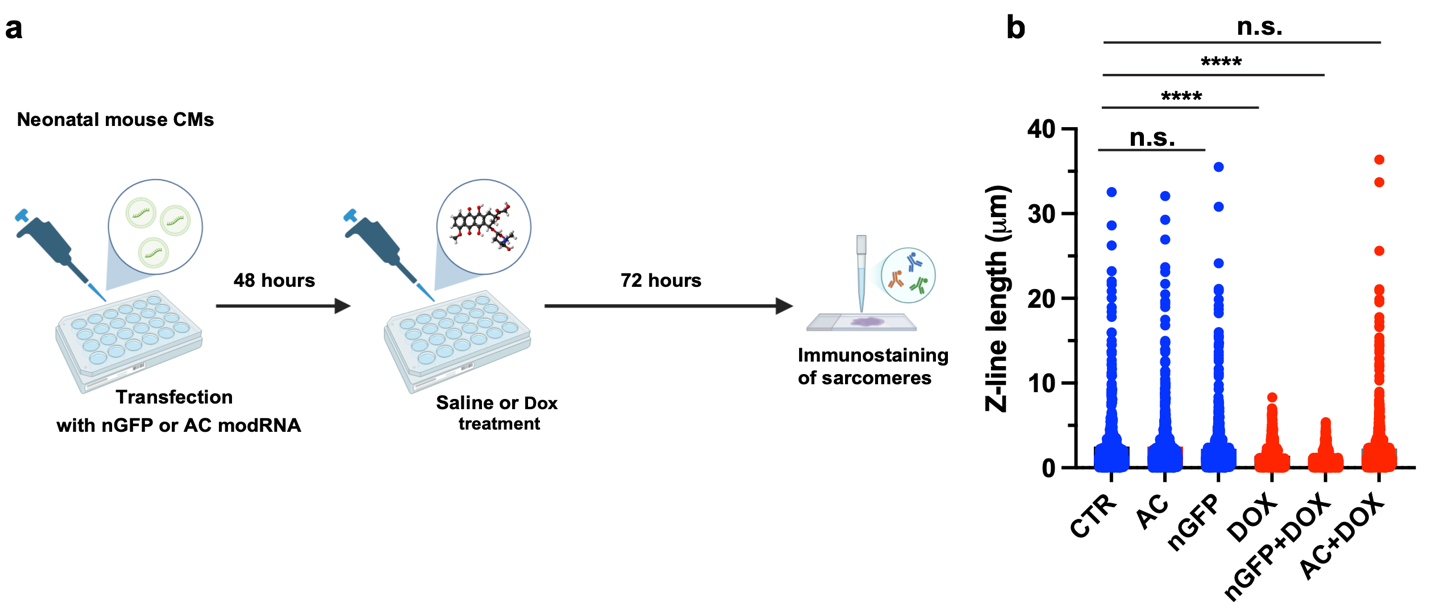
**

**Supplementary Figure 5. AC modRNA preserves sarcomere structure in neonatal mouse CMs treated with Dox.** **a**, Experimental timeline: Neonatal mouse CMs were isolated and transfected with either AC modRNA or control GFP modRNA. After 48 hours, cells were treated with 0.5 μM Dox for an additional 72 hours prior to analysis. **b**, Quantification of sarcomere length based on ACTN2 immunofluorescence staining (n = 700). Statistical analysis: One-way ANOVA with Tukey’s multiple comparison test. n.s., not significant; ****p < 0.0001.


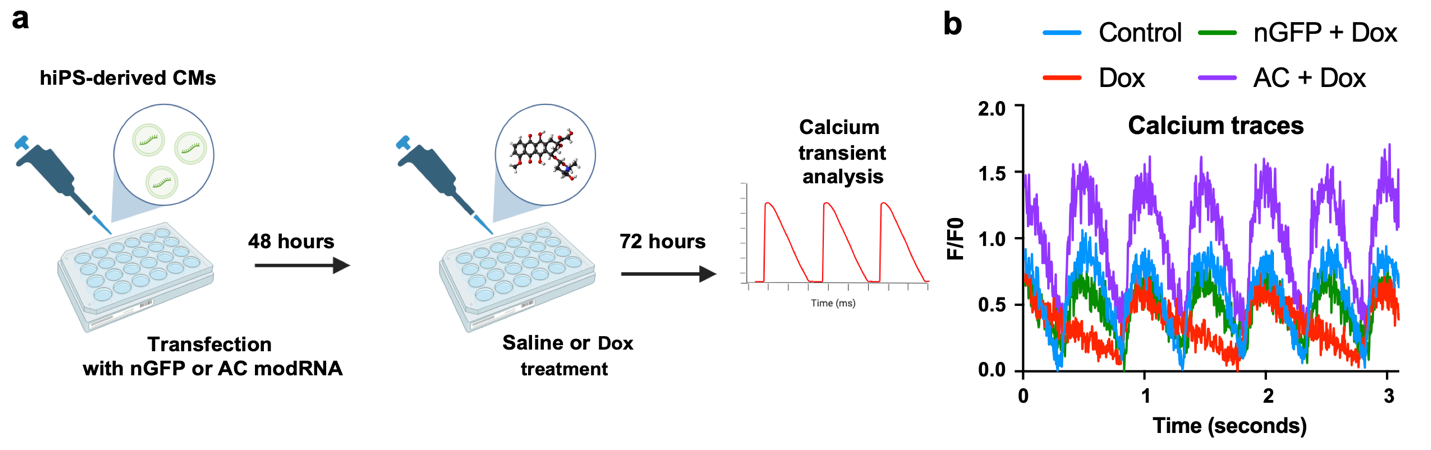


**Supplementary Figure 6. Acid ceramidase (AC) modRNA preserves calcium handling in hiPSC-CMs exposed to Dox.** **a**, Experimental workflow: hiPSC-CMs were transfected with AC or control GFP modRNA. After 48 hours, cells were treated with 0.5 μM Dox for 72 hours before functional and structural assays. **b**, A representative calcium trace for the average recording of calcium in cells exposed to different treatments. For quantification, please see Figure 2f. A fluorescence signal (F/F0, n = 754–1272) over time from primary cultured hiPS-derived CMs, indicating temporal cytoplasmic Ca2^+^ changes.

**
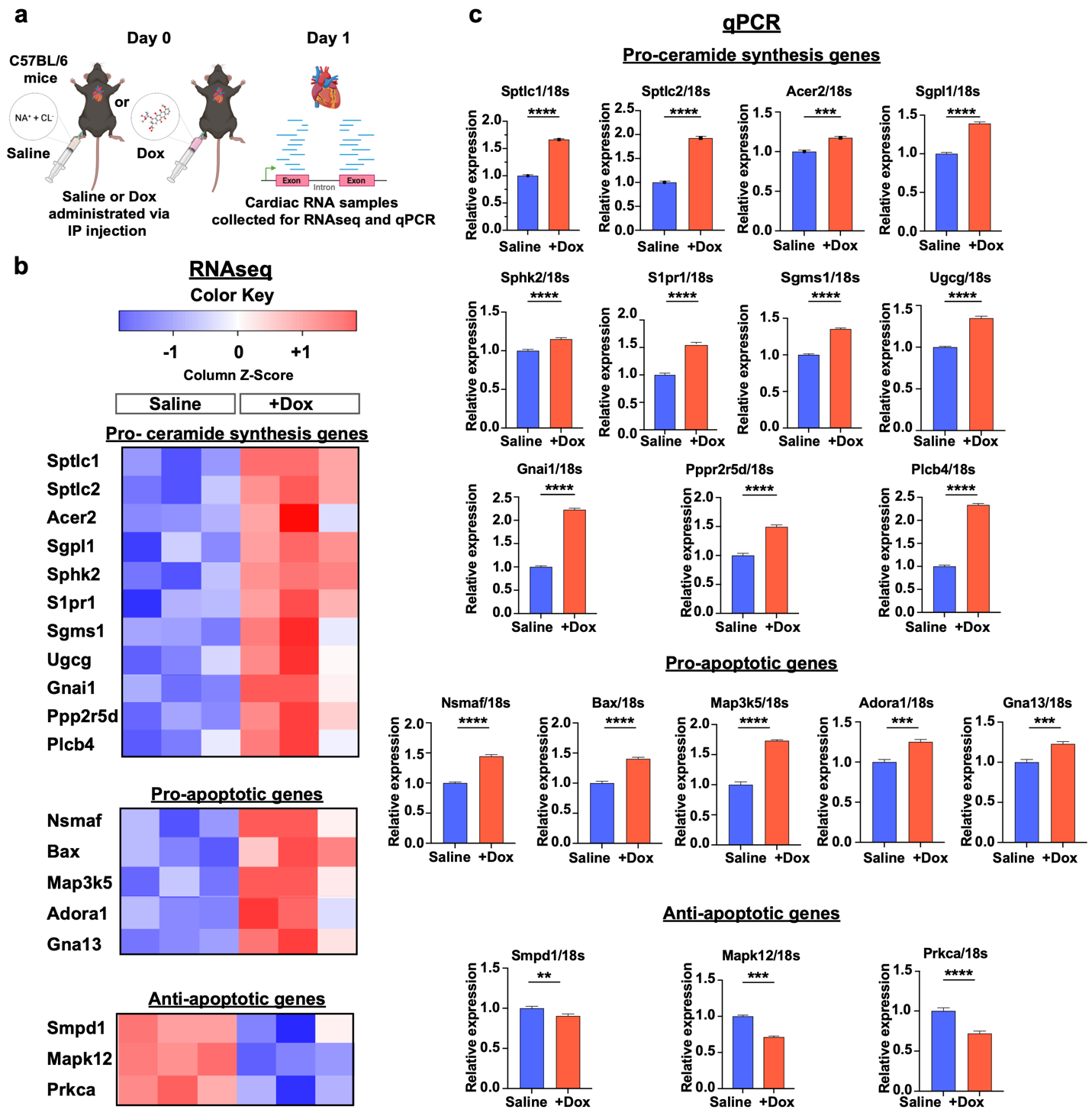
**

**Supplementary Figure 7. Dox treatment upregulates pro-ceramide synthesis and pro-apoptotic genes as well as downregulates anti-apoptotic genes in vivo.** **a**, Experimental timeline: Mice were treated with Dox or saline (control), and hearts were collected 24 hours later for gene expression analysis using RNA-seq and qPCR. **b**, RNA-seq analysis of gene expression changes in the heart following Dox treatment, compared to saline (n = 3). **c**, qPCR validation of RNA-seq findings (n = 3). Statistical analysis: Unpaired Student’s t-test. **p < 0.01; ***p < 0.001; ****p < 0.0001.

**
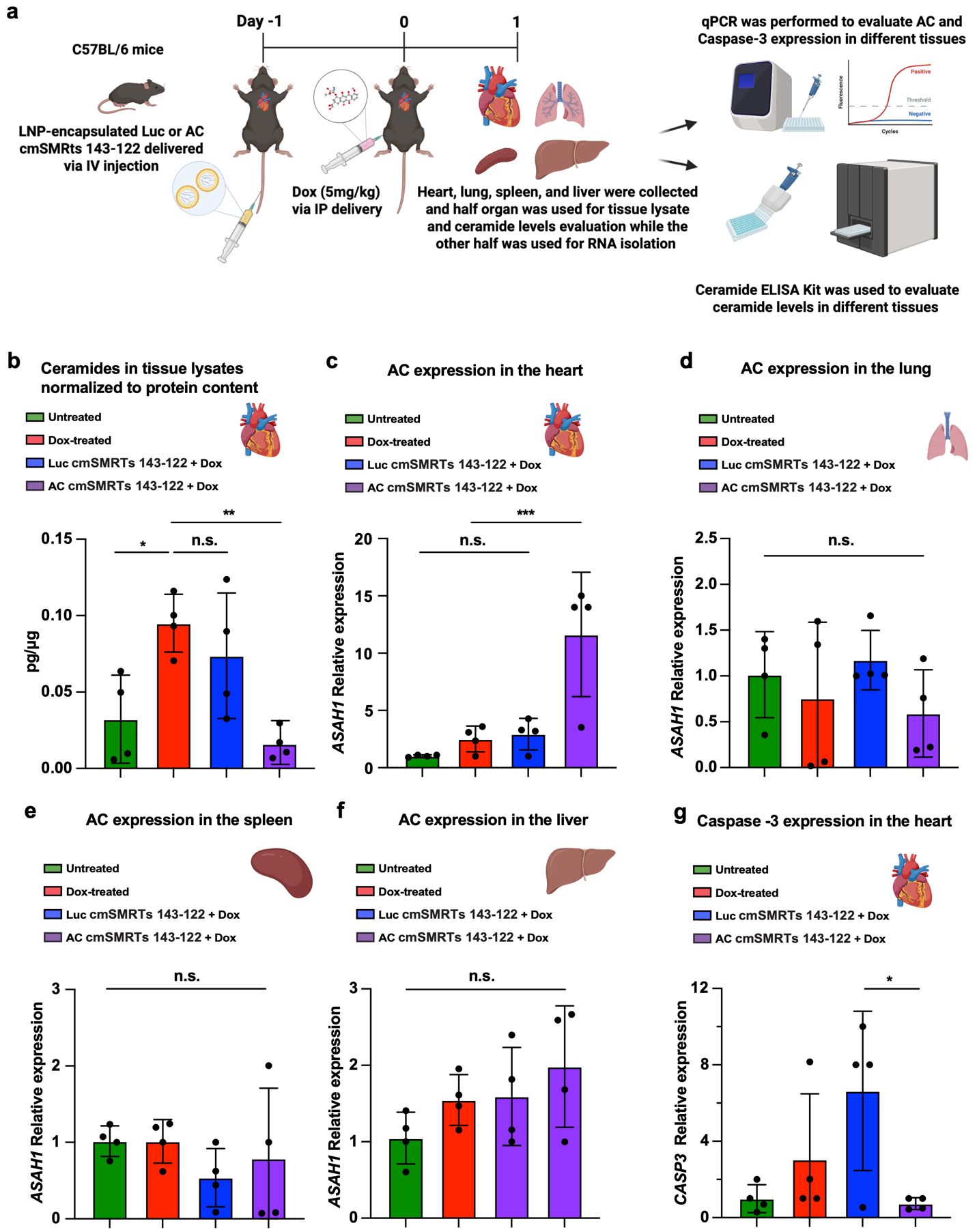
**

**Supplementary Figure 8. Systemic delivery of AC cmSMRTs 143-122 significantly reduces Dox-associated ceramide accumulation and apoptosis in the mouse heart in vivo.** **a**, Experimental timeline: C57BL/6 adult mice were pretreated (or not) with LNP-encapsulated Luc (control) or AC cmSMRTs 143-122, followed (or not) by IP injection of Dox (5 mg/kg). One day later, mice were sacrificed, and organs were collected. Ceramide ELISA kit was used to measure ceramide levels and qPCR assay was used to evaluate AC levels in the heart, liver, spleen, and lung as well as Caspase 3 levels in the heart. **b** Elisa quantification of ceramides levels in untreated group, Dox-treated group, and groups pretreated with either Luc or AC cmSMRTs 143-122. **c–f**, qPCR quantification of ASAH1 (AC) gene expression in different tissues after various treatments. **g,** Caspase-3 expression levels in the heart after different treatments. Statistical analysis: One-way ANOVA with Tukey’s multiple comparison test. n.s., not significant; *p < 0.05; **p < 0.01; ***p < 0.001.

**
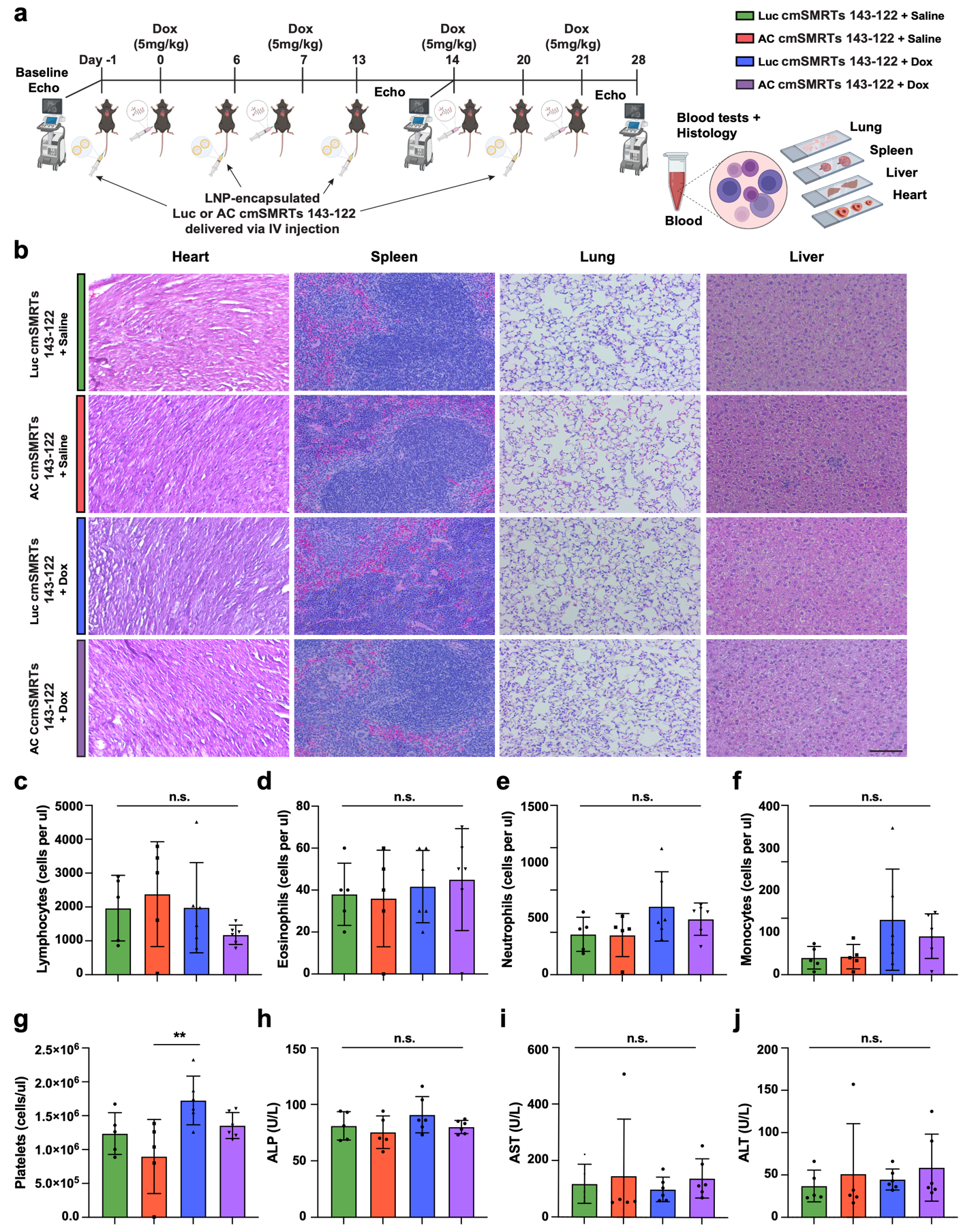
**

**Supplementary Figure 9. Systemic delivery of AC cmSMRTs 143-122 does not induce hematological or hepatic toxicity in adult male C57BL/6 mice.** **a**, Experimental timeline: Mice received weekly IV injections of LNP-encapsulated Luc or AC cmSMRTs 143-122, followed by IP administration of Dox (5 mg/kg) or saline (n = 5–6). **b**, Representative hematoxylin and eosin (H&E)-stained sections of heart, spleen, lung, and liver from treated mice, used to assess tissue integrity and the presence of bleeding or damage. Experiments were independently replicated twice. **c–g**, Complete blood count (CBC) analysis of lymphocytes (**c**), eosinophils (**d**), neutrophils (**e**), monocytes (**f**), and platelets (**g**) to evaluate immune responses across treatment groups. **h–j**, Quantification of liver enzyme levels, including of alkaline phosphatase (ALP, **h**), aspartate aminotransferase (AST, **i**), and alanine aminotransferase (ALT, **j**). Statistical analysis: One-way ANOVA with Tukey’s multiple comparison test. Scale bar in **b** = 40 µm. n.s., not significant; **p < 0.01.

**
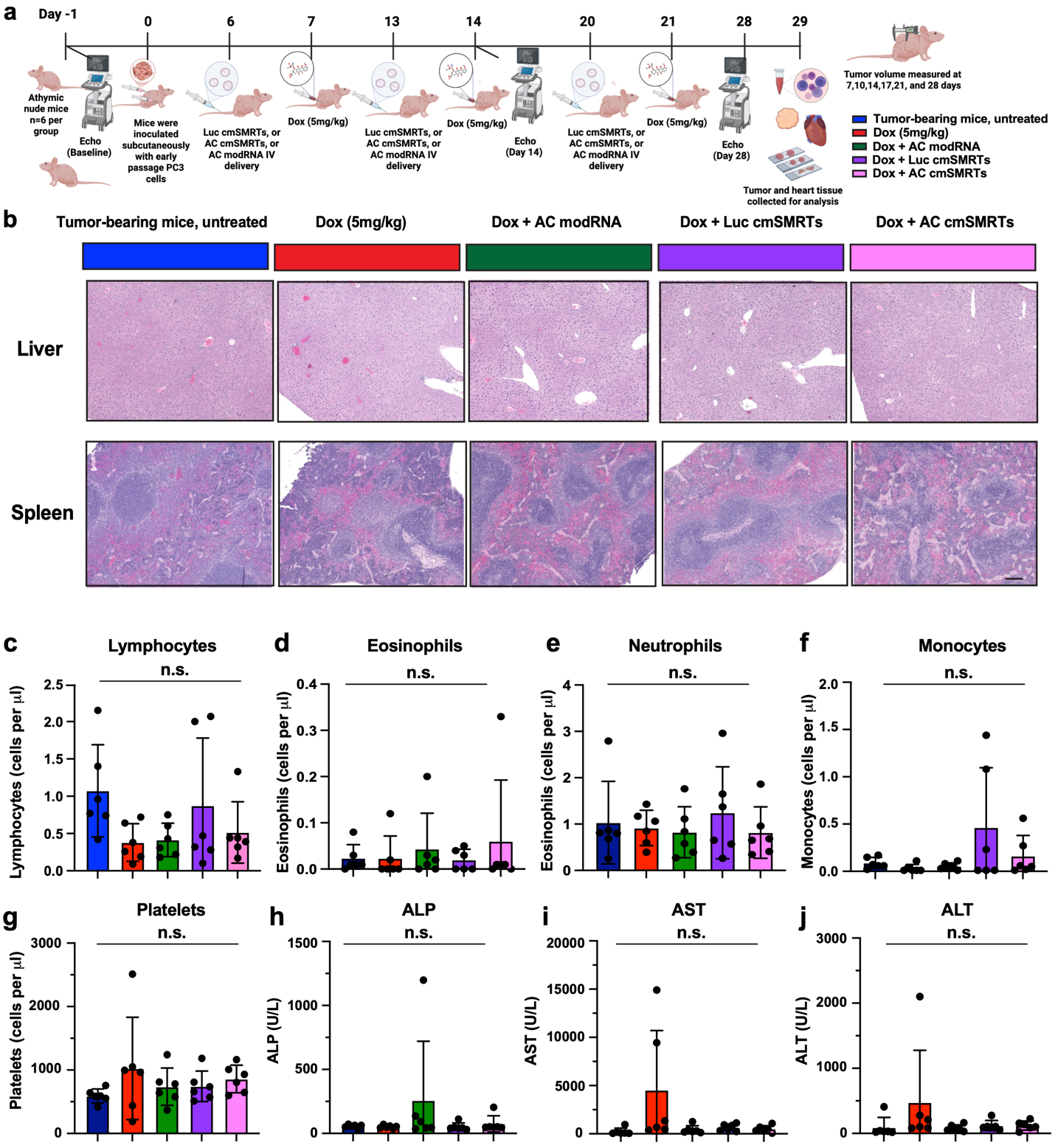
**

**Supplementary Figure 10. Systemic delivery of AC cmSMRTs 143-122 does not induce hematological or hepatic toxicity in adult male athymic nude mice bearing human prostate carcinoma (PC3) tumors. a**, Experimental timeline: Tumor-bearing male athymic nude mice (n = 6) were divided into five groups. One group remained untreated (Tumor-bearing mice, untreated). The second group received Dox treatment (5mg/kg) weekly at days 7, 14, and 21. The other three groups were pretreated with IV injections of LNP-encapsulated Luc cmSMRTs 143-122 (Dox + Luc cmSMRTs 143-122), AC modRNA (Dox + AC modRNA), or cmSMRTs 143-122 (Dox + Ac cmSMRTs 143-122) on days 6, 13, and 20. One day after each injection, mice received IP-injected Dox (5 mg/kg). Echocardiography was performed on days -1, 14, and 28. On day 29, hearts, tumors, tibias, and blood were collected to assess cardiac atrophy, complete blood count, and liver enzyme levels. **b**, Representative hematoxylin and eosin (H&E)-stained sections of liver and spleen from treated mice, used to assess tissue integrity and the presence of bleeding or damage. **c–g**, Complete blood count (CBC) analysis of lymphocytes (**c**), eosinophils (**d**), neutrophils (**e**), monocytes (**f**), and platelets (**g**) to evaluate immune responses across treatment groups. **h–j**, Quantification of liver enzyme levels, including of alkaline phosphatase (ALP, **h**), aspartate aminotransferase (AST, **i**), and alanine aminotransferase (ALT, **j**). Statistical analysis: One-way ANOVA with Tukey’s multiple comparison test. Scale bar in **b** = 50 µm. n.s., not significant; **p < 0.01.

**
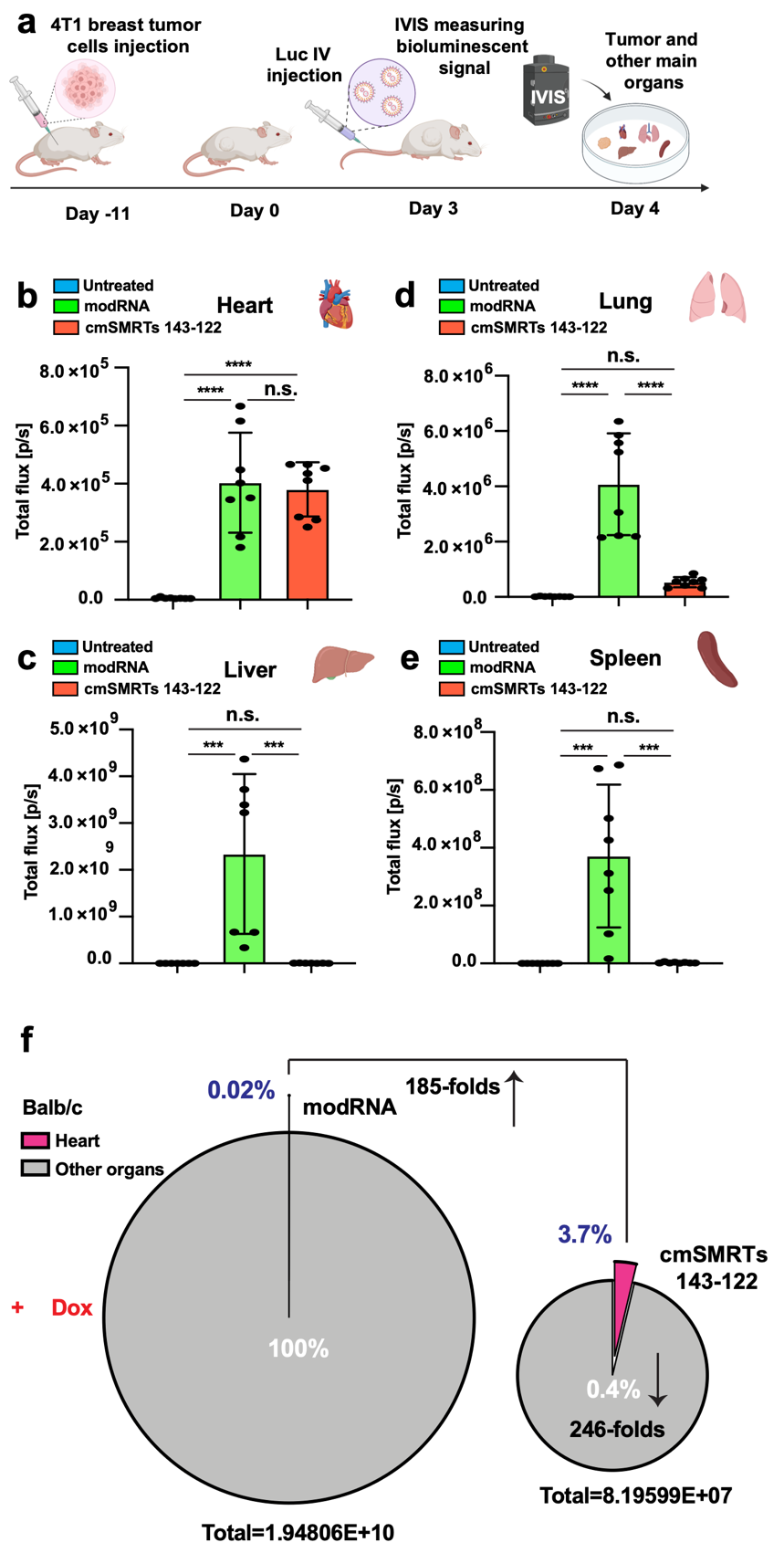
**

**Supplementary Figure 11. Tissue-specific expression following IV delivery of Luc modRNA or cmSMRTs 143-122 in 4T1 tumor-bearing Balb/c mice.** **a**, Experimental timeline: Balb/c mice bearing 4T1 breast tumors received IV injections of LNP-formulated Luc modRNA or cmSMRTs 143-122. Bioluminescence imaging (IVIS) was performed 24 hours post-injection to assess Luc expression in tumors and major organs. **b–e**, Quantification of Luc signal in the heart (**b**), liver (**c**), lung (**d**), and spleen (**e**) after IV delivery (n = 8). **f**, Pie chart comparing the distribution of total bioluminescence signal following IV delivery of Luc modRNA or cmSMRTs 143-122 in Dox-treated mice. Percentages in blue indicate the proportion of total expression localized in the heart. Percentages in white represent heart-specific expression relative to conventional Luc modRNA. Total luminescence values for each condition are listed beneath each chart. Statistical analysis: One-way ANOVA with Tukey’s multiple comparison test was used for **b–e**. n.s., not significant; ***p < 0.001; ****p < 0.0001.

**
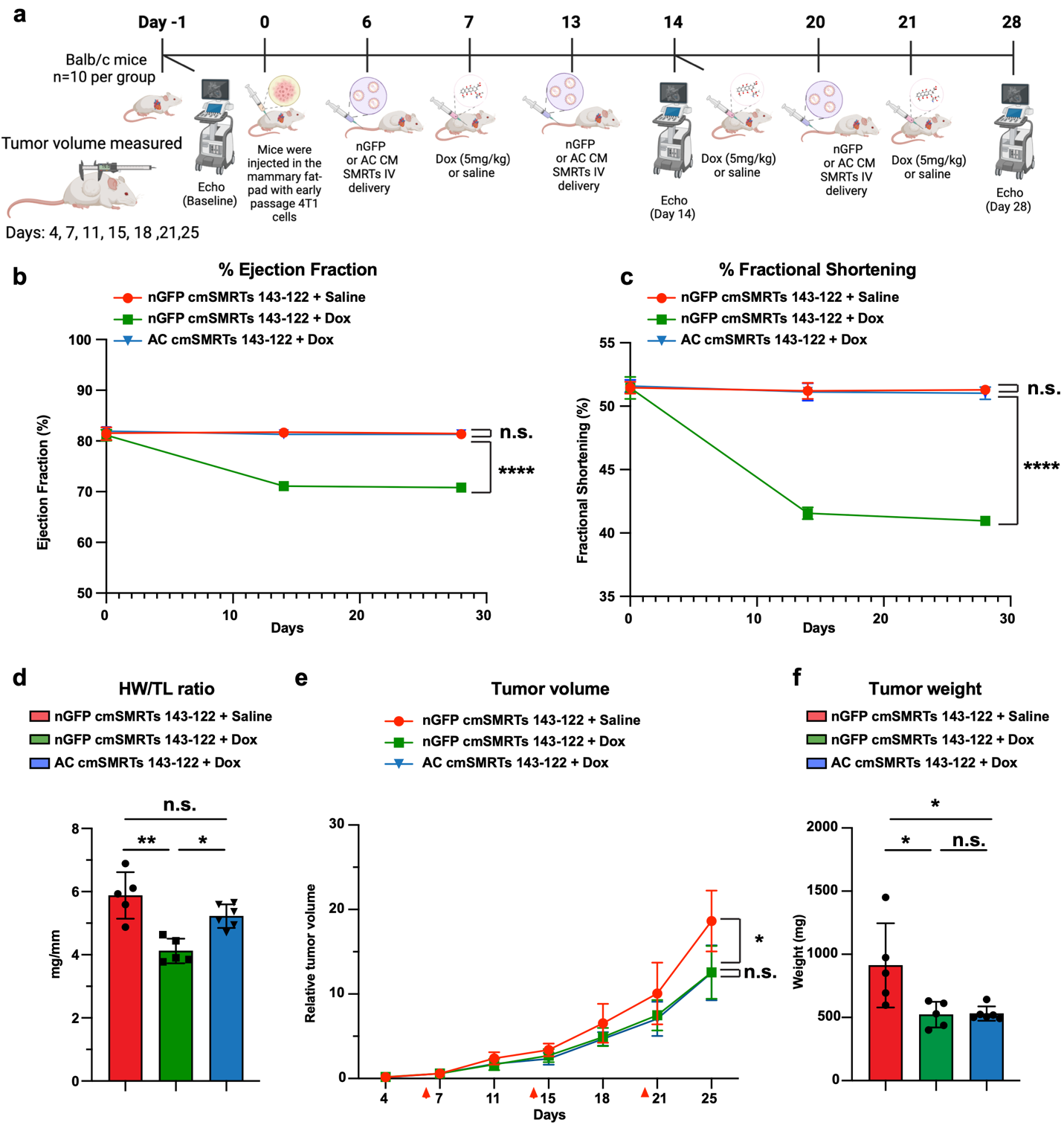
**

**Supplementary Figure 12. Pretreatment with AC cmSMRTs 143-122 prevents Dox-induced cardiotoxicity without compromising anti-tumor efficacy in female Balb/c mice bearing 4T1 breast tumors.** **a**, Experimental timeline: Tumor-bearing Balb/c mice received IV injections of LNP-encapsulated nGFP or AC cmSMRTs 143-122 on days 6, 13, and 20. One day after each injection, mice were treated with IP injections of saline or Dox (5 mg/kg). Echocardiography was performed on days -1, 14, and 28. On day 28, hearts, tibias, and blood were collected to assess cardiac atrophy, complete blood count, and liver enzyme levels (n = 5). **b–c**, Echocardiographic assessment of cardiac function showing % ejection fraction (**b**) and % fractional shortening (**c**) at the indicated time points. **d,** Quantification of heart-weight-to-tibia-length (HW/TL) ratio to assess cardiac atrophy at experimental endpoint (day 28). **e,** Relative tumor volume measurements over time for each treatment group. **f**, Tumor weight at experimental endpoint (day 28). Statistical analysis: Two-way ANOVA was used for **b–c and e**. One-way ANOVA with Tukey’s multiple comparison test was used for **d and f**; n.s., not significant; *p < 0.05; **p < 0.01; ****p < 0.0001.


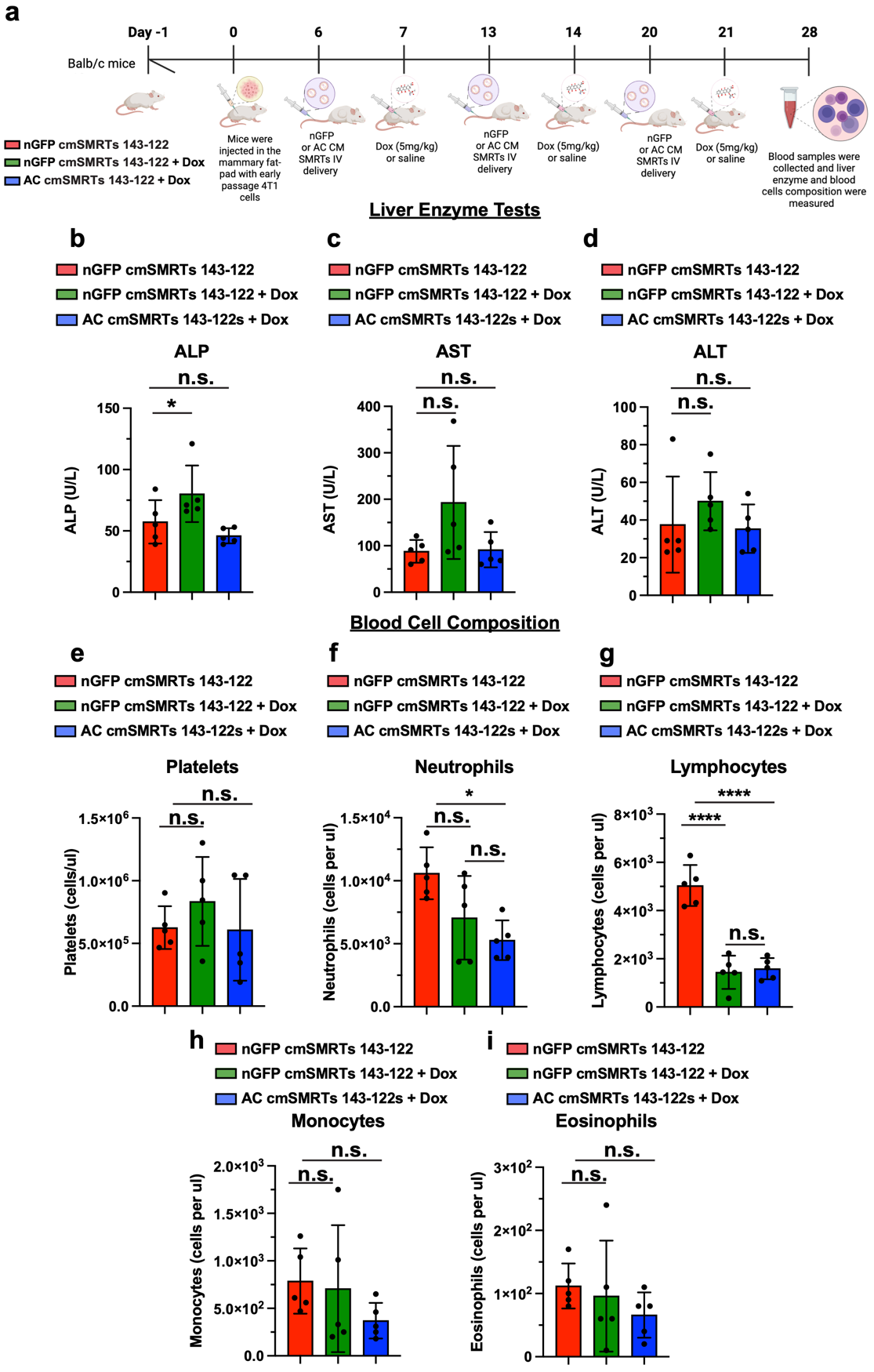


**Supplementary Figure 13. Systemic delivery of AC cmSMRTs 143-122 does not induce hematological or hepatic toxicity in 4T1 tumor-bearing Balb/c mice.** **a**, Experimental timeline: Balb/c mice bearing 4T1 breast tumors received weekly IV injections of LNP-encapsulated nGFP or AC cmSMRTs 143-122, along with IP injections of Dox (5 mg/kg). Mice treated with nGFP cmSMRTs 143-122 alone (without Dox) served as controls (n = 5). **b–d**, Serum liver enzyme levels were measured to assess hepatic toxicity: alkaline phosphatase (ALP, **b**), aspartate aminotransferase (AST, **c**), and alanine aminotransferase (ALT, **d**). **e–i**, Complete blood count analysis to evaluate systemic immune response, including platelet (**e**), neutrophil (**f**), lymphocyte (**g**), monocyte (**h**), and eosinophil (**i**) levels. Statistical analysis: One-way ANOVA with Tukey’s multiple comparison test. n.s., not significant; *p < 0.05; ****p < 0.0001.

**2) Supplemental Tables**

Table S1: Open reading frame sequences used for modRNA production

| Gene name | Sequence |
| --- | --- |
| Luc | atggccgatgctaagaacattaagaagggccctgctcccttctaccctctggaggatggcaccgctggcgagcagctgcacaaggccatgaagaggtatgccctggtgcctggcaccattgccttcaccgatgcccacattgaggtggacatcacctatgccgagtacttcgagatgtctgtgcgcctggccgaggccatgaagaggtacggcctgaacaccaaccaccgcatcgtggtgtgctctgagaactctctgcagttcttcatgccagtgctgggcgccctgttcatcggagtggccgtggcccctgctaacgacatttacaacgagcgcgagctgctgaacagcatgggcatttctcagcctaccgtggtgttcgtgtctaagaagggcctgcagaagatcctgaacgtgcagaagaagctgcctatcatccagaagatcatcatcatggactctaagaccgactaccagggcttccagagcatgtacacattcgtgacatctcatctgcctcctggcttcaacgagtacgacttcgtgccagagtctttcgacagggacaaaaccattgccctgatcatgaacagctctgggtctaccggcctgcctaagggcgtggccctgcctcatcgcaccgcctgtgtgcgcttctctcacgcccgcgaccctattttcggcaaccagatcatccccgacaccgctattctgagcgtggtgccattccaccacggcttcggcatgttcaccaccctgggctacctgatttgcggctttcgggtggtgctgatgtaccgcttcgaggaggagctgttcctgcgcagcctgcaagactacaaaattcagtctgccctgctggtgccaaccctgttcagcttcttcgctaagagcaccctgatcgacaagtacgacctgtctaacctgcacgagattgcctctggcggcgccccactgtctaaggaggtgggcgaagccgtggccaagcgctttcatctgccaggcatccgccagggctacggcctgaccgagacaaccagcgccattctgattaccccagagggcgacgacaagcctggcgccgtgggcaaggtggtgccattcttcgaggccaaggtggtggacctggacaccggcaagaccctgggagtgaaccagcgcggcgagctgtgtgtgcgcggccctatgattatgtccggctacgtgaataaccctgaggccacaaacgccctgatcgacaaggacggctggctgcactctggcgacattgcctactgggacgaggacgagcacttcttcatcgtggaccgcctgaagtctctgatcaagtacaagggctaccaggtggccccagccgagctggagtctatcctgctgcagcaccctaacattttcgacgccggagtggccggcctgcccgacgacgatgccggcgagctgcctgccgccgtcgtcgtgctggaacacggcaagaccatgaccgagaaggagatcgtggactatgtggccagccaggtgacaaccgccaagaagctgcgcggcggagtggtgttcgtggacgaggtgcccaagggcctgaccggcaagctggacgcccgcaagatccgcgagatcctgatcaaggctaagaaaggcggcaagatcgccgtgtaa |
| Asah1 | atgccgggccggagttgcgtcgccttagtcctcctggctgccgccgtcagctgtgccgtcgcgcagcacgcgccgccgtggacagaggactgcagaaaatcaacctatcctccttcaggaccaacgtacagaggtgcagttccatggtacaccataaatcttgacttaccaccctacaaaagatggcatgaattgatgcttgacaaggcaccagtgctaaaggttatagtgaattctctgaagaatatgataaatacattcgtgccaagtggaaaaattatgcaggtggtggatgaaaaattgcctggcctacttggcaactttcctggcccttttgaagaggaaatgaagggtattgccgctgttactgatatacctttaggagagattatttcattcaatattttttatgaattatttaccatttgtacttcaatagtagcagaagacaaaaaaggtcatctaatacatgggagaaacatggattttggagtatttcttgggtggaacataaataatgatacctgggtcataactgagcaactaaaacctttaacagtgaatttggatttccaaagaaacaacaaaactgtcttcaaggcttcaagctttgctggctatgtgggcatgttaacaggattcaaaccaggactgttcagtcttacactgaatgaacgtttcagtataaatggtggttatctgggtattctagaatggattctgggaaagaaagatgtcatgtggatagggttcctcactagaacagttctggaaaatagcacaagttatgaagaagccaagaatttattgaccaagaccaagatattggccccagcctactttatcctgggaggcaaccagtctggggaaggttgtgtgattacacgagacagaaaggaatcattggatgtatatgaactcgatgctaagcagggtagatggtatgtggtacaaacaaattatgaccgttggaaacatcccttcttccttgatgatcgcagaacgcctgcaaagatgtgtctgaaccgcaccagccaagagaatatctcatttgaaaccatgtatgatgtcctgtcaacaaaacctgtcctcaacaagctgaccgtatacacaaccttgatagatgttaccaaaggtcaattcgaaacttacctgcgggactgccctgacccttgtataggttggtga |
| Cre | Atgtccaatttactgaccgtacaccaaaatttgcctgcattaccggtcgatgcaacgagtgatgaggttcgcaagaacctgatggacatgttcagggatcgccaggcgttttctgagcatacctggaaaatgcttctgtccgtttgccggtcgtgggcggcatggtgcaagttgaataaccggaaatggtttcccgcagaacctgaagatgttcgcgattatcttctatatcttcaggcgcgcggtctggcagtaaaaactatccagcaacatttgggccagctaaacatgcttcatcgtcggtccgggctgccacgaccaagtgacagcaatgctgtttcactggttatgcggcggatccgaaaagaaaacgttgatgccggtgaacgtgcaaaacaggctctagcgttcgaacgcactgatttcgaccaggttcgttcactcatggaaaatagcgatcgctgccaggatatacgtaatctggcatttctggggattgcttataacaccctgttacgtatagccgaaattgccaggatcagggttaaagatatctcacgtactgacggtgggagaatgttaatccatattggcagaacgaaaacgctggttagcaccgcaggtgtagagaaggcacttagcctgggggtaactaaactggtcgagcgatggatttccgtctctggtgtagctgatgatccgaataactacctgttttgccgggtcagaaaaaatggtgttgccgcgccatctgccaccagccagctatcaactcgcgccctggaagggatttttgaagcaactcatcgattgatttacggcgctaaggatgactctggtcagagatacctggcctggtctggacacagtgcccgtgtcggagccgcgcgagatatggcccgcgctggagtttcaataccggagatcatgcaagctggtggctggaccaatgtaaatattgtcatgaactatatccgtaacctggatagtgaaacaggggcaatggtgcgcctgctagaagatggcgattag |
| Cas6 | atggaccactacctcgacattcgcttgcgaccggacccggaatttcccccggcgcaactcatgagcgtgctcttcggcaagctccaccaggccctggtggcacagggcggggacaggatcggcgtgagcttccccgacctcgacgaaagccgctcccggctgggcgagcgcctgcgcattcatgcctcggcggacgaccttcgtgccctgctcgcccggccctggctggaagggttgcgggaccatctgcaattcggagaaccggcagtcgtgcctcaccccacaccgtaccgtcaggtcagtcgggttcaggcgaaaagcaatccggaacgcctgcggcggcggctcatgcgccggcacgatctgagtgaggaggaggctcggaaacgcattcccgatacggtcgcgagagccttggacctgcccttcgtcacgctacgcagccagagcaccggacagcacttccgtctcttcatccgccacgggccgttgcaggtgacggcagaggaaggaggattcacctgttacgggttgagcaaaggaggtttcgttccctggttctga |
| nGFP | atggtgagcaagggcgaggagctgttcaccggggtggtgcccatcctggtcgagctggacggcgacgtaaacggccacaagttcagcgtgtccggcgagggcgagggcgatgccacctacggcaagctgaccctgaagttcatctgcaccaccggcaagctgcccgtgccctggcccaccctcgtgaccaccctgacctacggcgtgcagtgcttcagccgctaccccgaccacatgaagcagcacgacttcttcaagtccgccatgcccgaaggctacgtccaggagcgcaccatcttcttcaaggacgacggcaactacaagacccgcgccgaggtgaagttcgagggcgacaccctggtgaaccgcatcgagctgaagggcatcgacttcaaggaggacggcaacatcctggggcacaagctggagtacaactacaacagccacaacgtctatatcatggccgacaagcagaagaacggcatcaaggtgaacttcaagatccgccacaacatcgaggacggcagcgtgcagctcgccgaccactaccagcagaacacccccatcggcgacggccccgtgctgctgcccgacaaccactacctgagcacccagtccgccctgagcaaagaccccaacgagaagcgcgatcacatggtcctgctggagttcgtgaccgccgccgggatcactctcggcatggacgagctgtacaagggagatccaaaaaagaagagaaaggtaggcgatccaaaaaagaagagaaaggtaggtgatccaaaaaagaagagaaaggtataa |
| Nuclear mCherry | atggtgagcaagggcgaggaggataacatggccatcatcaaggagttcatgcgcttcaaggtgcacatggagggctccgtgaacggccacgagttcgagatcgagggcgagggcgagggccgcccctacgagggcacccagaccgccaagctgaaggtgaccaagggtggccccctgcccttcgcctgggacatcctgtcccctcagttcatgtacggctccaaggcctacgtgaagcaccccgccgacatccccgactacttgaagctgtccttccccgagggcttcaagtgggagcgcgtgatgaacttcgaggacggcggcgtggtgaccgtgacccaggactcctccctgcaggacggcgagttcatctacaaggtgaagctgcgcggcaccaacttcccctccgacggccccgtaatgcagaagaagaccatgggctgggaggcctcctccgagcggatgtaccccgaggacggcgccctgaagggcgagatcaagcagaggctgaagctgaaggacggcggccactacgacgctgaggtcaagaccacctacaaggccaagaagcccgtgcagctgcccggcgcctacaacgtcaacatcaagttggacatcacctcccacaacgaggactacaccatcgtggaacagtacgaacgcgccgagggccgccactccaccggcggcatggacgagctgtacaagtaa |

Table S2: Primer sequences for qPCR

| **Gene** | **Forward** | **Reverse** |
| --- | --- | --- |
| 18s | AGTCCCTGCCCTTTGTACACA | CGATCCGAGGGCCTCACTA |
| Sptlc1 | GAGGAGTCACCGAGCACTATGG | GCTGATGGTCAACCACGAAGGA |
| Sptlc2 | CCAGACTGTCAGGAGCAACCAT | CTTCTTGTCCGAGGCTGACCAT |
| Acer2 | GAGGACAACTACACTATCGTGCC | TAGATGCCGCTGTTGAAGCACG |
| Sgpl1 | GGAAAGCCTCAGGAGCTGTGTA | CTGCCTCTAACTTCCGCAATCC |
| Sphk2 | GGTGCCAATGATCTCTGAAGCTG | CTCCAGACACAGTGACAATGCC |
| S1pr1 | CGCAGTTCTGAGAAGTCTCTGG | GGATGTCACAGGTCTTCGCCTT |
| Sgms1 | GCATAGTTGGCACGCTGTACCT | TAAGCCACCTCCAGCAATGAGC |
| Ugcg | CCGTATGTAGCCGACAGACAAG | CCCGTCACACATTTGAAGCCAG |
| Gnai1 | CTCGGAAGAGGAGTGTAAGCAG | GCAAGCACGAAAAGTTGGCGAG |
| Ppp2r5d | GAACACAAGGTGTTTCTCGTCCG | TCCTTCTCCAGGAACTGCACCA |
| Plcb4 | CAGTCACAAGGAAGCGGTTGCA | GGATGTTCGTGGTAGCACCTAC |
| Nsmaf | GGATCTGGGAAAGAGACAAGGC | CGTAACCGCTTTCCAAGGCATC |
| Bax | AGGATGCGTCCACCAAGAAGCT | TCCGTGTCCACGTCAGCAATCA |
| Map3k5 | GGTCATTCAGGCATCCGAGAAG | CAGAAGTCCACGAGTTCCTGCT |
| Adora1 | GATCGGTACCTCCGAGTCAAGA | CACTCAGGTTGTTCCAGCCAAAC |
| Gna13 | TCCACCTTCCTGAAGCAGATGC | AGCTTCTCTCGGGCATCTACCA |
| Smpd1 | AACTCTGAGCCGACCACTAGCT | GTCCAGGACCACATGAGAGCTT |
| Mapk12 | GGCTACTGGATGTGTTCACACC | CTGGATTCTGTCTTCACTCAGGG |
| Prkca | ACAACCTGGACAGAGTGAAACTC | CTTGATGGCGTACAGTTCCTCC |
| Asah1 | ACAGGATTCAAACCAGGACTGT | TGGGCATCTTTCCTTCCGAA |
| Gapdh | AGGTCGGTGTGAACGGATTTG | TGTAGACCATGTAGTTGAGGTCA |

Table S3: List of immunostaining antibodies

| **Antibody Name** | **Company** | **Catalog number** |
| --- | --- | --- |
| ACTN2 | Abcam | Ab9465 |
| TNNT2 | Abcam | Ab91605 |
| tdTomato | Sicgen | AB8181 |
| Vimentin | Invitrogen | MA5-11883 |
